# Pre-training in Medical Data: A Survey

**DOI:** 10.1007/s11633-022-1382-8

**Published:** 2023-02-21

**Authors:** Yixuan Qiu, Feng Lin, Weitong Chen, Miao Xu

**Affiliations:** 1 The University of Queensland, Brisbane, 4072 Australia; 2 The University of Adelaide, Adelaide, 5005 Australia

**Keywords:** Medical data, pre-training, transfer learning, self-supervised learning, medical image data, electrocardiograms (ECG) data

## Abstract

Medical data refers to health-related information associated with regular patient care or as part of a clinical trial program. There are many categories of such data, such as clinical imaging data, bio-signal data, electronic health records (EHR), and multi-modality medical data. With the development of deep neural networks in the last decade, the emerging pre-training paradigm has become dominant in that it has significantly improved machine learning methods′ performance in a data-limited scenario. In recent years, studies of pre-training in the medical domain have achieved significant progress. To summarize these technology advancements, this work provides a comprehensive survey of recent advances for pre-training on several major types of medical data. In this survey, we summarize a large number of related publications and the existing benchmarking in the medical domain. Especially, the survey briefly describes how some pre-training methods are applied to or developed for medical data. From a data-driven perspective, we examine the extensive use of pre-training in many medical scenarios. Moreover, based on the summary of recent pre-training studies, we identify several challenges in this field to provide insights for future studies.

## 1 Introduction

Artificial intelligence (AI) has become a tremendously ubiquitous technique impacting our lives. Applications based on artificial intelligence assist users in making decisions and influencing their daily lives. Technological advances are not possible without the rapid development of deep learning (DL), especially thanks to a much wider adoption of convolutional neural network (CNN)[1, 2], recurrent neural network (RNN)[3, 4], and attention neural network[5, 6]. Those deep neural networks have been integrated into a variety of research, including several subfields such as computer vision (CV)[7] and natural language processing (NLP)[8].

Medical data analysis is one of the most important sub-filed in AI. The task mainly focuses on processing and analysing the medical data from variant data modalities to extract essential information which aims to help physicians make precise decisions during the diagnosis process. It is anticipated that computer-aided systems will be influential tools in health monitoring and disease diagnosis. A lot of efforts have been successful in current studies, such as processing and analysing medical imaging[9–11], electronic health records (EHRs)[12, 13], bio-signals[14–16] and the data which consists of multiple modalities[17–20]. Hou et al.[21–23] utilised CNN to diagnose tumours in the early stages, allowing for early intervention treatment planning to greatly improve the patient's survival rate. A medicine recommendation[12, 24] was developed as a way to improve patient care by providing personalized recommendations based on electronic health records. Qiu et al.[14] supported caregivers in identifying cardiac arrhythmias effectively and efficiently, saving more lives. Wang et al.[17] utilised chest X-rays and the corresponding diagnosis reports training a model for disease diagnosis, similarity search, and image regeneration.

Although existing works have achieved remarkable success, some works found that data-hungry is one of the primary challenges of applying the DNN for processing medical data. On the one hand, some kind of medical data can be obtained easily, but annotating the collected data requires a substantial amount of labour and money; on another hand, in many rare or new disease diagnosis tasks, the data is insufficient because they are too rare to collect or there are issues in privacy. The insufficient data have limited training for a satisfactory model because it could cause overfitting and poor generalization. To address this issue, some large-scale datasets are proposed to make it possible to train satisfactory models. However, the construction of large-scale annotated datasets is labor-consuming and expensive. It is unpractical to develop large-scale annotated datasets.

The researchers, motivated by human learning

Review
Special Issue on Large-scale Pre-training: Date, Models, and Fine-tuning
Manuscript received June 30, 2022; accepted October 18, 2022; published online February 21, 2023
Recommended by Associate Editor Jun-Zhou Huang
©The Author(s) 2023

strategy, proposed the pre-training to address the issue of lack of annotated data. Considering the human learning strategy, learners can learn a skill based on their prior learning knowledge. For example, learning to play tennis can help in learning badminton.

As summarized in [25], the pre-training technique is specially related to transfer and self-supervised learning. As one of the most critical milestones for solving data-hungry issues, transfer learning techniques have explored utilising labelled data and leveraging the unlabelled data effectively. Transfer learning[26] is a sub-field of machine learning inspired by the process of human learning. It learns the related knowledge in the target domain by transferring information from the same or related domain[27]. The process of transfer learning consists of two steps, pre-training and fine-tuning. Pre-training is a process of learning universal feature representations and then using the pre-trained model in the downstream tasks, as Fig. 1 shows. The recently emerging self-supervised learning is another pre-training paradigm which gets wide notice by more and more researchers. This learning paradigm is committed to extracting abundant knowledge from unlabelled data. Self-supervised learning enables the production of the supervision information by itself instead of manual annotations. In the current studying stage, transfer learning and self-supervised learning are two mainstream pre-training approaches. In this paper, we introduce these two approaches at a high-level and explore pre-training in the medical domain.

## 1.1 Why pre-training?

However, the emergence of pre-training, mainly including transfer learning and self-supervised learning, provides the opportunity to use a small size of labelled data to train an effective model in the efficient method. In this section, we list the reasons why pre-training is essential. Firstly, the pre-training method was invented from the lack of data information, which is generally divided into the lack of labels and the lack of data volume[28]. The lack of data volume means that many types of data cannot meet the needs of model training, such as very scarce regional rare disease data. Pre-training can effectively compensate for the impact of this lack of information[28]. Through pre-training, clusters or potential features in the data are extracted by the model so that the model has more generalization ability for specific content.

Secondly, the utilization of pre-trained models can significantly accelerate the convergence process on downstream tasks. This is particularly beneficial in scenarios where computational resources are constrained.

Thirdly, in the past 20 years, with the rapid development of various industries and the generation of high-performance hardware, a large amount of data has been rapidly generated daily in different industries, such as the medical industry[29]. However, the cost of manual annotation of datasets increases exponentially. Therefore, the supervised pre-training methods have challenges on the lack of data annotation. Self-supervised pre-training allows us to leverage abundant non-labelled data, getting a good initialization before the downstream tasks.

Also, with the recent advance in self-supervised learning, many studies[30, 31] have shown that self-supervised pre-training can alleviate the effect of training on data with imbalanced labels.

There are also many applications of pre-training in the medical field. Pre-training technology was first implemented in the medical domain in 2014 by Schlegl et al.[32], in which they proposed a semi-supervised learning approach to improve lung tissue classification. Specifically, they train the pre-training model with the unsupervised strategy injecting the information from the images without annotation. There are three modalities of the data that we mainly focus on that have been processed with pre-training successfully: medical image data, biosignal, and EHR. Also, the multi-modality scenario has

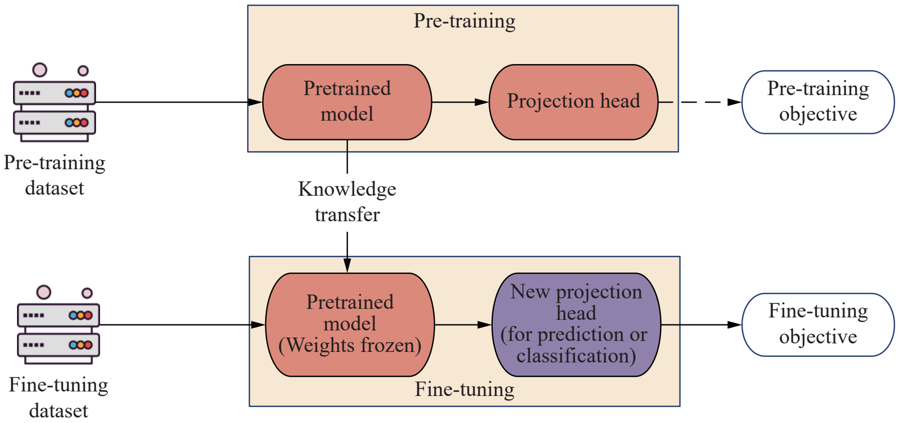

Fig. 1 Illustration of pre-training. Pre-training is a part of transfer learning. If the pre-training model is fully supervised, the pre-training objectives are required, while if the pre-training model is an unsupervised or self-supervised learning model, the pre-training process does not need the objective.

been considered. For example, the pre-trained BERT model in semantic analysis can be applied to predict future diagnoses using EHR data[33]. The self-supervised pre-trained model can perform tasks such as classification and segmentation on CT and MRI images[34]. The electrical bio-signals can be pre-trained to extract the features, thereby helping to perform prediction or diagnosis[35]. These pre-training applications in the medical field improve the performance of many tasks. Compared with conventional models, pre-training has significantly improved efficiency and accuracy in medical field applications.

## 1.2 Why is this survey necessary?

There are two reasons why we have organised this survey. First, many works using pre-trained models have achieved satisfactory results in the medical domain in the past few years, but there are few systematic and comprehensive introductions to pre-training models. Second, [25] is a comprehensive survey for pre-training, while there is no such a survey about pre-training in the medical domain. The existing surveys[36–39] in the medical field only focus on investigating the pre-training models for the specific modality. Particularly, most surveys about pre-training in the medical domain are to review pre-training in medical imaging[36, 37], and few surveys are published for reviewing processing biosignals[40] and EHRs[38]. Therefore, it is significant that we systematically review pre-training approaches in the medical domain.

To the best of our knowledge, this paper is the first systematic and comprehensive summary of the recent pre-training innovations in the medical field, consisting of medical imaging analysis, electric bio-signal data (electroencebhalograms (EEG), electrocardiograms (ECG) etc.), EHRs and multi-modality.

This survey presents the techniques and analysis in a simple manner, which is suitable for a variety of audiences. However, we emphasise the core target audience of the survey mainly for two groups. One group has experts from the medical field who are interested in developing a computer-aided diagnosis system. An additional perspective reader is an expert in machine learning and deep learning and wants to learn about the current developments in pre-training in medicine.

## 1.3 Collection of paper

We summarize the survey strategy from the bibliographic dataset, keywords of searching conduction, and the main focus on the papers that have been published in conferences/journals.

In this survey, we retrieved papers purely related to pre-training in the medical domain. The related papers retrieving were executed on four well-known bibliography websites, including Google scholar, DBLP, ACM digital library and Web of Science. To collect all the papers possible, we initially searched for the terms "transfer learning/pre-training/self-supervised/contrastive learning" + "medical data/medical images/bio-signal data/EHR/multi-modality/prognosis". In particular, we pay close attention to top-ranking conferences/journals, including CVPR/ICCV, MICCAI, IJCAI, KDD, ICDM, AAAI, WWW, NeurIPS, ICML, TPAMI, TMI, MIA, *Nature*, *Science*, etc. Furthermore, we also screen the results of other conferences, journal papers, and preprint versions on arXiv to ensure that this survey is more comprehensive. We also reviewed many surveys that investigated the pre-training in image processing tasks and NLP-related tasks. As among the collection papers and the former survey, most of them have introduced the basic models, like CNN, RNN, Transformer, self-supervised learning, etc., we will not re-visit these basic techniques and not review the specific papers that introduce the related techniques theoretically. In a particular scenario, model pre-training is usually inextricably linked to the downstream task, including fine-tuning or training a classifier for a particular task.

## 1.4 Our contributions

This survey aims to present a systematic introduction to recent advances and new frontiers of pre-training-based techniques in the medical domain. We summarized more than 200 advanced contributions in this field using pre-training technology, covering the time range from the very beginning of the emergence of pre-training approaches. We list several main contributions of this survey.

1) We first systematically summarized the pre-training techniques that are used for medical and clinical scenarios.

2) We summarized the medical pre-training models used on four main data types: medical images, bio-signal data, EHR data, and multi-modality. To our best knowledge, we are the first to do a survey so comprehensively.

3) We summarized the benchmark dataset of medical images, bio-signal and EHRs.

4) We discuss the challenges of the pre-training model in the medical domain and look to the topics for future research.

The rest of this survey is structured as follows. Section 2 briefly introduces the benchmark datasets in the medical domain and the basic models and methods for pre-training. Section 3 summarises pre-training on medical imaging analysis for different datasets. Section 4 gives an introduction to pre-training for bio-signal. Section 5 summarises the state-of-the-art pre-training methods for EHRs. In Section 6, we discuss the challenges and future directions. Finally, Section 8 gives the conclusion of the survey.

## 2 Background

This section will summarise the publicly available benchmark dataset in the medical domain. Moreover, some basic pre-train methods are briefly introduced.

### 2.1 Benchmark datasets

In this section, we extensively explore the benchmark datasets which can be used in machine learning (ML) and DL-based tasks in the medical domain.

#### 2.1.1 Medical imaging benchmarking datasets

Computer vision has been a popular topic in medical imaging processing. There are hundreds of datasets in this field. As listed in Table 1, this study presents a comprehensive overview of 16 frequently utilised publicly accessible medical imaging datasets. Table 1 includes the name of each dataset, the modalities they encompass, their potential applications, and benchmarking results.

#### 2.1.2 Bio-signal medical benchmark datasets

As listed in Table 2, we summarize 22 bio-signal benchmark datasets that are publicly available or have access restrictions. We present the modalities in the dataset, the number of subjects (# Subjects), the number of records (# Records), the sampling rate, the related task, and the comparison of the results.

#### 2.1.3 EHRs benchmark datasets

Based on the survey, we only found four publicly available benchmark datasets are reusable for EHRs related tasks, such as the eICU Collaborative Research Database (eICU)[41], the Medical Information Mart for Intensive Care III (MIMIC III)[42], IQVIA[1], and PhisoNet Challenge 2012[2][43]. In some works, they use their private dataset to execute the experiments. In the following, we

Table 1 Statistics of the medical imaging benchmark datasets. Here, SEG is the abbreviation of "Segment", and CLS denotes "Classification". In the column "# of images", the number shows the ratio of number of the training set to number of the testing set.

| Dataset name | Modality | # of images | # of classes | Tasks | Benchmarking results (%) |
|---|---|---|---|---|---|
| CHAOS-MRI[44] | MRI | 992 | 5 | SGE | Dice-socre: 86.75; MSD: 66.00[45] |
| CHAOS-CT[44] | CT | 2 874 | 2 | SEG | Dice-score: 97.79 ±0.43;<br>MSSD: 21.89±13.94[44] |
| NIH-CT-82[46] | CT | 7 141 | 2 | SEG | Dice: 71.80±10.70[46] |
| MICCAI 2017 LiTS[47] | CT | 131/70 | 2 | SEG | Lesion: Dice: 82.40<br>Liver: Dice: 96.50[48] |
| 3Dircadb* | CT | 20 venous phase | 2 | SEG | Tumour: Dice: 93.70 ± 0.20[48] |
| OCT2017[49] | CT | 207 130 | 4 | CLS | Acc.: 92.80; Sen: 93.20; Spe: 90.10[49] |
| BUSI[50] | Ultrasound | 780 | 3 | CLS | The original tumour image (ROI):<br>Acc: 88.46; F1: 83.33; AUC:90.52<br>The segmented tumour image:<br>Acc: 90.77; F1:84.21; AUC:88.57<br>The tumour shape image (TSI):<br>Acc: 85.38; F1: 75.95; AUC:86.60<br>The fused image:<br>Acc: 94.62; F1: 91.14; AUC:97.11[51] |
| CheXpert[52] | X-ray | 224 316 | 14 | CLS | Avg. Acc on 14 categories: 90.70[52] |
| ChestXray 14[18] | X-ray | 112 120 | 14 | CLS | AUROC: 84.40[52] |
| EyePACS† | Fundoscopic | 35 126 | 5 | CLS | Acc: 91.10; AUC: 95.70[53] |
| ISIC2019[54] | Dermoscopy | 25 331 | 9 | CLS | AUC: MEL: 92.80; NV: 96.00;<br>BCC: 94.90; AK: 91.40; BKL: 90.4;<br>DF: 97.9; VASC: 95.60; SCC: 93.80;<br>UNK: 77.5[55] |
| TCGA-GBM[56] | WSI | 255 | – | SA | C-index: 64.52[57] |
| TCGA-LGG[56] | WSI | 1 061 | – | SA | C-index: 74.10[58] |
| TCGA-LUSC[56] | WSI | 485 | – | SA | C-index: 62.87[57] |
| NLST[59] | WSI | 1 104 | – | SA | C-index: 64.76; AUC: 66.93[60] |
| PatchCamelyon‡ | WSI | 262 144/32 768 | – | SA | Acc: 89.80; AUC: 96.30; NLL: 26.00[61] |

* https://www.ircad.fr/research/data-sets/liver-segmentation-3d-ircadb-01/
† https://www.kaggle.com/c/diabetic-retinopathy-detection/
‡ https://github.com/basveeling/pcam

1 https://www.iqvia.com/insights/the-iqvia-institute
2 https://physionet.org/content/challenge-2012/1.0.0/

Table 2 Statistics of the bio-signal benchmark datasets. Here, AD is the abbreviation of arrhythmia diagnosis, SSD denotes sleep-state detection, SD denotes seizure detection, ED means emotion detection.

| Name | Modalities | # Records | # of subjects | Task | Sampling rate | Benchmarking results (%) |
|---|---|---|---|---|---|---|
| MIT-BIH arrhythmia dataset[62] | ECG | 48 | 1 | AD | 360 Hz | CNN: Acc: 92.56; F1: 92.54; AUC: 99.20[14] |
| PTB-XL[63] | ECG | 21 837 | 71 | AD | 500 Hz | AUC: 92.90 Deep learning for ECG analysis: benchmarks and insights from PTB-XL |
| MIT-BIH noise stress test[64] | ECG | 15 | 1 | Denoise | 360 Hz | CNN: Acc: 96.19; F1: 96.18; AUC: 99.64[14] |
| European ST-T database[65] | ECG | 90 | 2 | AD | 250 Hz | CNN: Acc: 92.53; F1: 91.06; AUC: 99.20[14] |
| AF classification challenge 2017[66] | ECG | 8 528 | 4 | AD | 300 Hz | F1: 72.00[67] |
| PTB diagnostic ECG[68] | ECG | 549 | 9 | AD | N/A | Acc: 96.00; Pre: 99.00; Re: 93.00 using 12 leads[69] |
| AHA[70] | ECG | 154 | 8 | AD | 250 Hz | CNN: ACC: 99.70; F1: 99.71; AUC: 99.98[14] |
| CPSC2018[71] | ECG | 6 877 | 8 | AD | 500 Hz | Overall F1: 84.00[72] |
| AMIGOS[73] | ECG, GSR | N/A | 40 | ED | 128 Hz | Arounsal(Acc: 76.00); Valence(Acc: 75.00)[74] |
| ASCERTAIN[75] | EEG, ECG, EDA | N/A | 58 | ED | N/A | [75] |
| BIO-VID-EMO DB[76] | ECG, EMG, SC | N/A | 86 | ED | N/A | Acc: 79.51[77] |
| DEAP[78] | EEG, EDA, EMG, PPG, EOG, RSP | N/A | 32 | ED | 512 Hz | Arousal(Acc: 77.19, F1:69.01); Valence(Acc: 76.17; 72.43)[79] |
| DREAMER[80] | EEG, ECG | N/A | 23 | ED | ECG:256 Hz | Valence(Acc: 86.23); Arousal(Acc: 84.54); Dominance(85.02)[81] |
| MAHNOB-HCI[82] | EEG, ECG, EDA, RSP, SKT | N/A | 27 | ED | N/A | $M_e(SD_e)$: –3.30(6.88) bpm; RMSE: 7.62 bpm; $M_{eRate}$: 6.87[83] |
| MPED[84] | EEG, ECG, EDA, RSP, EEG | N/A | 23 | ED | N/A | [84] |
| SEED[85] | EEG | N/A | 15 | ED | 200 Hz | Acc: 85.65[85] |
| Temple University Hospital (TUH)[86] | EEG | N/A | 315 | SD | 200 Hz | Classification error rate: 20.66 |
| Sleep-EDF: Telemetry[87] | EEG, EOG, EMG | N/A | 22 | SSD | 100 Hz | Acc: 82.00; MF1: 76.90; $\kappa$: 76.00[88] |
| MASS-1* | EEG, EOG, EMG | N/A | 53 | SSD | 256 Hz | Acc 86.20; MF1: 81.70; $\kappa$:80.00[88] |
| SHHS[89, 90] | EEG, EOG, EMG | N/A | 5 804 | SSD | 12 550 Hz | Pre: 86.00%; Rec: 87.00; Spe: 95.00[91] |

Note: The benchmark results of datasets, ASCERTAIN and MPED, are hard to show in the table. Check them in the original paper.
* https://ceams-carsm.ca/en/MASS/

briefly introduce these four datasets.

1) MIMIC-III contains all patients admitted to the intensive care unit (ICU) at Beth Israel Deaconess Medical Center from 2001 to 2012 and includes over 60 000 unique ICU admissions with millions of observations.

2) eICU is a multi-center database comprised of identified health data that contains over 200 000 ICU admissions across the United States between 2014 and 2015.

3) IQVIA is a real-world patient and clinical trial database that can be requested from the website[3]. This dataset contains 2 609 clinical trials formed between 2014 and 2019, and includes 25 894 doctors across 28 countries.

4) Physionet Challenge 2012 contains 11 records and 988 adult ICU admissions. The dataset is used to predict in-hospital mortality given the first 48 hours of data for each ICU admissions.

[3] https://www.iqvia.com/insights/the-iqvia-institute

## 2.2 Pre-training

From a historical perspective, the term pre-training was first introduced in 2007 in the works of Bengio et al.[92, 93] They proposed a model which consisted of greedy layer-wise unsupervised pre-training followed by supervised fine-tuning. The pre-training techniques have been widely used after deep neural networks achieved success[25].

The research on deep neural networks (DNN) has encountered the bottleneck of a lack of training data (generally lack of annotated data). The problem comes from the fact that it is easy to encounter overfitting and poor generalization issues by training a DNN network with many parameters without sufficient training data[25]. To solve this issue, in the early stage, some researchers attempted to construct massive annotation datasets for AI tasks from the data level. However, data annotation is a time and cost-consuming task. Moreover, few AI-related tasks have a related large-scale annotated dataset for training, e.g., tasks in the medical domain. How to train a generalized and robust model for specific tasks with few annotated samples has attracted more attention for a long time.

Pre-trained models have achieved significant success in the AI community. We employ those pre-trained models as a backbone to get representative embeddings for downstream tasks. Generally, we adopt the pre-training technique in the following situations.

1) The training and pre-training datasets are related tasks[25, 94, 95]. If the source and target datasets are similar tasks or in the same domain, we can use the pre-trained model instead of training a model from scratch, which significantly improves efficiency.

2) Training datasets have an extremely small size of the annotated samples[25, 94]. The small size of the datasets cannot be sufficient to satisfy the training of a high-performance model. A pre-trained model can be introduced under this circumstance to improve the quality of the feature representation, thus getting a satisfactory performance in the downstream tasks.

3) The computation resource is limited[94]. The pre-trained models can speed up convergence on the target task, which allows models to converge sufficiently within fewer iterations. That is friendly for situations where the computation is limited.

4) The data samples are sufficient, but the labelling budget is small[29]. In the current era of explosive digital data growth, it is easy to collect massive unlabelled data, while annotating them is expensive. In this situation, a self-supervised learning paradigm will help with learning a generalized representation of the unlabelled data and then use such a model to process the downstream tasks.

We list four essential scenarios in which a pre-trained model will be considered to introduce. Though they are mentioned separately, they have overlapped parts, meaning there are no explicit application situations of pre-training. Pre-training can be widely used in multiple circumstances where the rich knowledge benefits various downstream tasks[25]. As [25] mentioned, pre-training is highly related to transfer learning and self-supervised learning. In the following sections, we will mainly focus on introducing these two paradigms in detail.

The different supervision levels can impact two phases in the pre-training: the pre-training itself and the downstream tasks using the pre-training results. In the pre-training phase, both supervised and unsupervised cases exist. For example, transfer learning could be either supervised or unsupervised, while self-supervised learning can be unsupervised. Whether pre-training methods should be adopted depends on the source data and target data′s relationship and the supervision level given in the target data. Generally speaking, the more similarity between the target domain and source domain, the more benefit of the pre-training methods; the less supervision information provided in the downstream task (such as semi-supervised learning), the more benefit will be provided by the pre-training methods.

#### 2.2.1 Transfer learning

Transfer learning is the primary strategy for early pre-training. It is a significant paradigm motivated by the human study process that learners study new knowledge based on previous knowledge. It mainly focuses on solving new problems through the influence of experience and knowledge in pre-training on the target tasks[25, 96]. This process enables us to use the already learned knowledge from the source domain freely and learn a new skill by accepting little knowledge and speeding up learning. Thus, reflecting the AI task, this process can be generalised to two learning steps: pre-training prior knowledge from the source task and fine-tuning to learn more specific knowledge from the target source[27]. In transfer learning, pre-training can be categorised into supervised and unsupervised pre-training based on the source data with supervision information or not. Whether the target data is labelled or not, the supervised pre-training can be divided into inductive and transduction transfer learning[27], as shown in Fig. 2. For transductive transfer learning, a classifier is trained with labelled data, using the pre-trained classifier to produce pseudo labels for the target domain dataset. The data with the produced pseudo labels would be added to the training set. An example of this training fashion is self-training[97], which is considered a specific semi-supervised learning technique.

Generally, feature transfer and parameter transfer are the two main goals in transfer learning pre-training[27]. For example, after pre-training a computer vision model on a large labelled dataset "ImageNet", a small amount of data can be fine-tuned to achieve reliable results because the features and parameters of the target task have been learned by the model in the pre-training of the large dataset. In addition, instance transfer and relational knowledge are two other pre-training approaches in transfer learning. Many feasible large-scale models were developed for pre-training, such as AlexNet[7], VGGNet[2], ResNet[98], GoogleNet[99], DenseNet[100], etc. In the same way, inspired by transfer learning in the CV domain, pre-training also is widely used in the NLP domain. The pre-trained word representation models extract word embeddings as an input of NLP tasks. There have been many

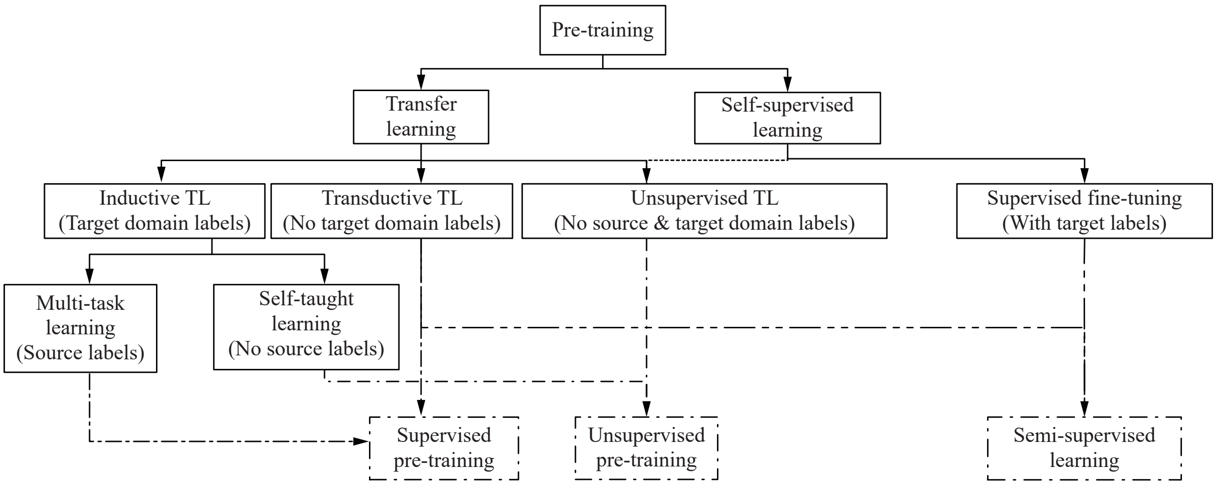

Fig. 2 Illustration of the relationship between pre-training and other methods (unsupervised, supervised, semi-supervised)

well-known pre-training models proposed in recent years, e.g., embeddings from language models (ELMo)[101] and the well-known pre-trained model BERT[8] that is trained on a large-scale dataset.

Transfer learning has been so influential in deep learning in recent years that it has become an integral approach to processing medical data. There have been a considerable number of works using transfer learning to improve the performance in medical imaging analysis, such as in radiology[18, 52], pathology[102], dermatology[103], ophthalmology[104, 105], etc. Most of these previous works were proposed based on a standard pipeline that introduces pre-trained ImageNet models to extract universal representations for various medical imaging modalities. For example, Wang et al.[18, 52, 106] initialized the weights of backbones from ImageNet pre-trained models. Esteva et al.[103] demonstrated a DNN-based diagnosis of skin lesions approach using the ImageNet pre-trained model as a feature extractor, getting a competitive performance with dermatologists. Treder et al.[105] utilised ImageNet pre-trained model to extract features for 1 112 spectral domain optical coherence tomography images, and Han et al.[102] utilised the same feature extraction method in the histopathology image classification and segmentation tasks.

Apart from the imaging modality, transfer learning has been successful in other data modalities, but there is no dataset as large as ImageNet for non-image medical data. Some works convert the one-dimensional vector into images with Fourier or Wavelet transformation and then use the ImageNet pre-trained models in feature extraction[107, 108]. Moreover, other works transfer the feature extractor between different but related tasks[16, 109]. In addition to these strategies, some researchers pre-trained feature extractor on one dataset, which contains relatively large samples, and then transferred the model to process other datasets that could be sparse and small[16, 110]. Clinical text data is an NLP-related task. Most currently proposed methods use the BERT model to extract word embeddings[111].

Although transfer learning has successfully processed many tasks, the conventional transfer learning paradigm still has controversial issues in the medical domain. A standard formulation for imaging data is using ImageNet pre-trained models. Matsoukas et al.[112] pointed out that transfer learning works by increasing the reuse of learned representations. However, there are many remarkable differences in data (including size, distribution, categories, etc.), features, and the final tasks between the natural classification task on ImageNet and the target specification medical data[95]. He et al.[94] refered to the fact that the ImageNet pre-trained model can help to speed up convergence but does not contribute to performance improvement. Especially in relatively large-scale medical datasets, the ImageNet pre-trained model has no obvious advantages compared to a simple model[95]. In addition, the systematical experiments show that the ImageNet pre-trained model is over-parameterized for medical image tasks instead of extracting more sophisticated features[95].

### 2.2.2 Self-supervised pre-training

Considering that there are a large number of data produced in the real world that are not annotated, to leverage such data, self-supervised learning has become one of the most promising ways of processing unlabelled data in deep learning[113–115]. It attempts to gain the supervisory signal from the data pool rather than human annotation, then exploits the underlying semantic information to learn general data representations for downstream tasks.

The typical workflow of self-supervised learning, similar to transfer learning, consists of representation and downstream task learning. The actual self-supervised learning happens in the first stage, where the model learns the knowledge of the unstructured dataset to represent the feature embeddings, which is the exact difference between self-supervised learning and conventional transfer learning. In the downstream learning process, the framework could be the same as in supervised fashion: a feature extractor followed by a classifier. The top feature extractor will be initialized using the weights transferred from the first stage, and the transferred weights will be fine-tuned as the particular task, training the following modules meanwhile. Another setting is after self-supervised learning without fine-tuning. The extracted features of the unlabelled training dataset will classify using clustering methods, like the k-nearest neighbours algorithm (KNN).

Based on the aforementioned, from the data-driven perspective, the self-supervised learning fashion is similar to the transfer learning settings with unsupervised pre-training: they are all trained on unlabelled data. The self-supervised learning can be regarded as a branch of transfer learning as a consequence. However, they are trained in different ways: unsupervised pre-training is generally trained on the model without supervision, e.g., clustering[116]; instead, self-supervised learning typically is trained through an end-to-end framework using the self-produced supervision information. Furthermore, the self-supervised learning approach can also be considered semi-supervised when the embeddings are fine-tuned with supervision[117]. The relationship is shown in Fig. 2.

Nowadays, various self-supervised learning frameworks have been developed and succeeded in many domains for applications, such as in the communities of CV and NLP, etc. For instance, many advanced frameworks firstly were developed for CV tasks, like CPC[118], momentum contrast (MoCo)[119], SimCLR[113], BYOL[114], SwAV[120], MAE[121], Siamese[122], etc. Additionally, BERT[8] still performs poorly in processing NLP-related tasks. For extended applications, self-supervised learning has become one of the best choices for processing medical data. The reason is that the amount of annotated data is relatively small, while the unlabelled data is considerably large in real-world medical datasets. Therefore, the remainder of this section will delve into the exploration of state-of-the-art self-supervised learning techniques.

To achieve better understanding, we will introduce readers to the state-of-the-art self-supervised learning methods in the upcoming sections.

Contrastive predictive coding (CPC) is an unsupervised contrastive learning approach to learning high-dimensional data representation, which can be used in many data modalities, such as text, speech and image data[118]. CPC aims to learn useful and informative representations that keep more information on the raw data and can precisely predict the future state latent vector. The architecture of the CPC model has two main components, including a non-linear encoder and an auto-regressive model. The illustrations of CPC frameworks are shown in Figs. 3 and 4, representing the architecture of processing time-series data and images respectively. The non-linear encoder ($g_{enc}$) maps the raw sequence to a lat-

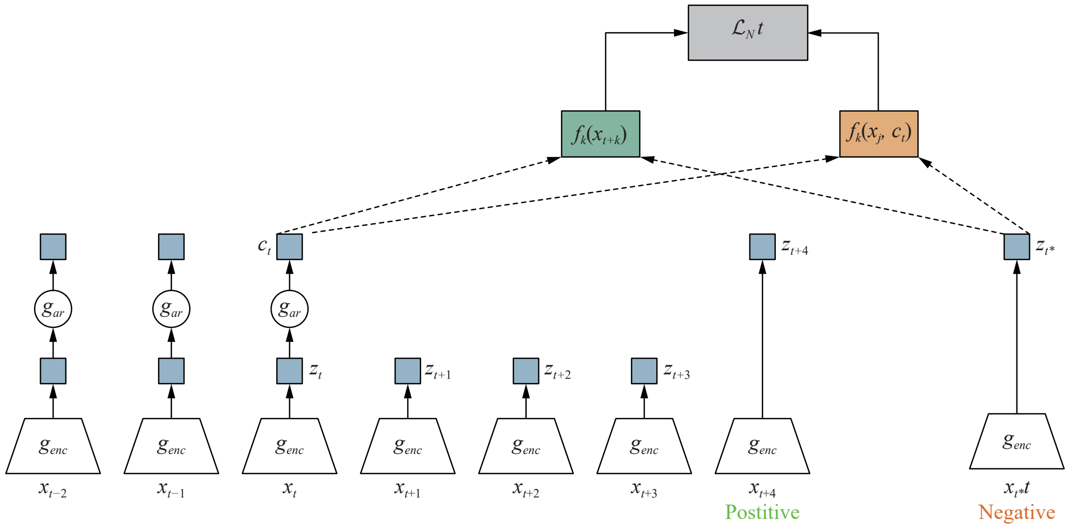

Fig. 3 Architecture of CPC for time-series data

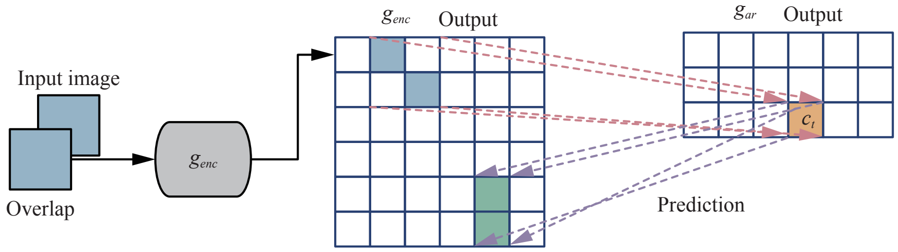

Fig. 4 Architecture of CPC for image data ent representation ($z_t = g_{enc}(x_t)$). The auto-regressive model, like GRU, as a predictor enables the prediction of the context latent representation ($c_t = g_{ar}(z_t)$), which condenses the information before the $t$ state. To evaluate the prediction, here, $c_t$ is used to predict the latent vector of the after-$k$ states. In practice, a linear transformation is used to obtain the predicted latent representation ($\widehat{z_k} = W_t c_t$), where $W_t$ is a learnable linear matrix. A score here defines the relativeness between the predictive representation and the real future sequence. The scoring function can be expressed as the following equation:

$$f_k(x_{t+k}, c_t) = \exp(z_{t+k}^{\mathrm{T}} W_k c_t). \tag{1}$$

The non-linear encoder and auto-regressive model are optimised jointly based on the noise-contrastive estimation (NCE) loss, namely, InfoNCE, in this work. The loss function is shown as follows:

$$\mathcal{L}_N = -E\left[\log \frac{f_k(x_{t+k}, c_t)}{\sum_{x_j \in X} f_k(x_j, c_t)}\right] \tag{2}$$

where the sequence set $X = x_1, x_2, \cdots, x_N$ in which has one positive sample, the other $N-1$ samples are regarded as negative samples[123].

MoCo[119] is a mechanism of contrastive learning intuited by dictionary look-up via a dynamic dictionary with a queue and moving-averaged encoder, as Fig. 5 shows. MoCo learns the robust representation via performing dictionary look-up by making the query embeddings closed and its matching key embedding far away. There are two necessities to build such a reasonable dictionary: 1) The dictionary should be large enough; 2) The representation should be consistent (the key requires to be encoded using a similar or identical encoder to be meaningful of the similarity metric between query and key in the dictionary). The proposed MoCo mechanism achieves the first necessity through a dynamic memory bank, where the oldest mini-batch will be progressively replaced by a new one. At the same time, a momentum update method is introduced to address the key representations′ consistency, as defined in (4) defined. The model will be optimized in MoCo with the InfoNCE loss, which is defined as the following equation:

$$\mathcal{L}_q = -\log \frac{\exp(f_q(x^q) f_{k_+}(x^{k_+})/\tau)}{\sum_{i=0}^{K} \exp(f_q(x^q) f_{k_i}(x^{k_i})/\tau)} \tag{3}$$

where $\tau$ is a temperature hyper-parameter that is used to smooth the loss, $f_q(x^q)$ denotes the query that is the encoded features and a set of embeddings $\{k_0, k_1, k_2, \cdots\}$ encoded with $f(\cdot)$. The encoded samples $k$s are the keys of a dictionary in which a single key $k_+$ matches $q$, and the $k_i$ is considered as the negative sample for $q$. Another important technique, the momentum update, is defined as

$$\theta_k \simeq m\theta_k + (1-m)\theta_q \tag{4}$$

where $\theta_k$ is the weight of the encoder $f_k(\cdot)$ and $\theta_q$ is regarded as $f_q(\cdot)$. $m \in [0, 1)$ is a momentum coefficient. A larger $m$ yields better performance, as the experiments show in [119]; when $m = 0.999$, the performance is best. In further research, the authors of MoCo proposed MoCo-v2[124] and MoCo-v3[125] to improve the performance.

Swapping assignments between multiple views (SwAV)[120] is a cluster assignment-based contrastive learning paradigm. The illustration of SwAV is shown in Fig. 6. Compared to the previous contrastive learning methods, SwAV does not calculate view-pair comparison, instead comparing the cluster assignments of the different views, thus not requiring huge computation resources. Apart from introducing a clustering mechanism, SwAV proposes a multi-crop augmentation strategy aiming to increase the number of image views while not burdening with extra memory and computation cost. Piratically, SwAV introduces online clustering, which maps the features ($Z = z_1, z_2, \cdots, z_B$) to a set of vectors ($Q = q_1, q_2, \cdots, q_B$) by the prototype ($C = c_1, c_2, \cdots, c_K$). Then, the newly defined loss is to minimize the similarity with a setup of swapped prediction. The loss shows as the following equation:

$$\mathcal{L}(z_t, z_s) = l(z_t, q_s) + l(z_s, q_t) \tag{5}$$

where the $l(\cdot)$ is described as the following equation. Here, we give an example with $l(z_t, q_s)$.

$$\ell(\boldsymbol{z}_t, \boldsymbol{q}_s) = -\sum_k \boldsymbol{q}_s^{(k)} \log \boldsymbol{p}_t^{(k)}$$

where

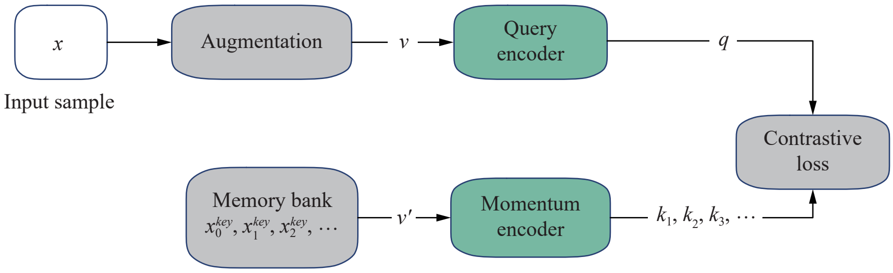

Fig. 5 Illustration of MoCo

$$\boldsymbol{p}_t^{(k)} = \frac{\exp\left(\frac{1}{\tau}\boldsymbol{z}_t^{\mathrm{T}}\boldsymbol{c}_k\right)}{\sum_{k'}\exp\left(\frac{1}{\tau}\boldsymbol{z}_t^{\mathrm{T}}\boldsymbol{c}_{k'}\right)}. \tag{6}$$

Simple framework for contrastive learning of visual representations (SimCLR)[113] is a straightforward implemented framework for contrastive learning, which was first proposed to process image data, yielding state-of-the-art performance. The framework of SimCLR is shown in Fig. 7. It learns representation on an unlabelled dataset by maximizing agreement between random conducting augmentation methods for the same data sample via a contrastive loss. Similarly, the same data was augmented with two randomly selected methods producing two views. These two views are treated as a cheerful pair, while considering all other samples in the same batch as the negative samples. The augmented views pass through the backbone, typically a large-scale neural network, producing the embeddings that are the features of the data we want to get via the pre-training settings. The dimensionality of trained embeddings is reduced through a multi-layer non-linear projection head. The losses are calculated by conducting the loss function for a cheerful pair and its corresponding negative samples in a mini-batch. The contrastive loss function is defined as

$$\mathcal{L}_{CL,i} = -\log\frac{\exp(s_{i,j}^{+}/\tau)}{\exp(s_{i,j}^{+}/\tau)+\sum Q_{[i\neq k]}\exp(s_{i,k}^{-}/\tau)} \tag{7}$$

where $s^{+}$ is the matrix of the similarity between one positive pair $(z_i, z_j)$, $s^{-}$ is the matrix of the similarity between the negative pairs $(z_i, z_k)$, $\tau$ represents the temperature parameter used to smooth the labels, and $Q$ means the distribution of the mini-batch in the current state. The similarity matrix could be calculated with $sim(u,v) = \boldsymbol{u}^{\mathrm{T}}\boldsymbol{v}/\|\boldsymbol{u}\|\|\boldsymbol{v}\|$.

Bootstrap your own latent (BYOL)[114] is another popular approach to self-supervised representation learning. The framework of BYOL is shown in Fig. 8. Unlike the SimCLR and MoCo[119], BYOL can learn high-quality representations relying on only one augmented view of an image, which means that it does not require negative samples. Particularly, the framework consists of two neural networks to learn the representation, where an online network is trained to predict the representations produced by the target network. In this way, the additional predictor module in the online network can prevent the model's collapse. The loss function can be defined as the following mean square error (MSE) between the predic-

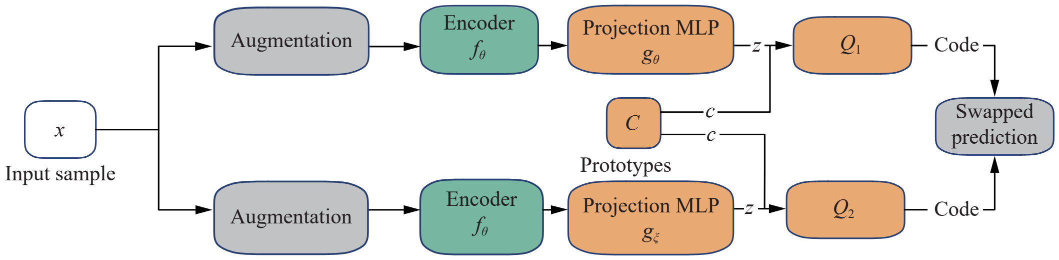

Fig. 6 Illustration of SwAV

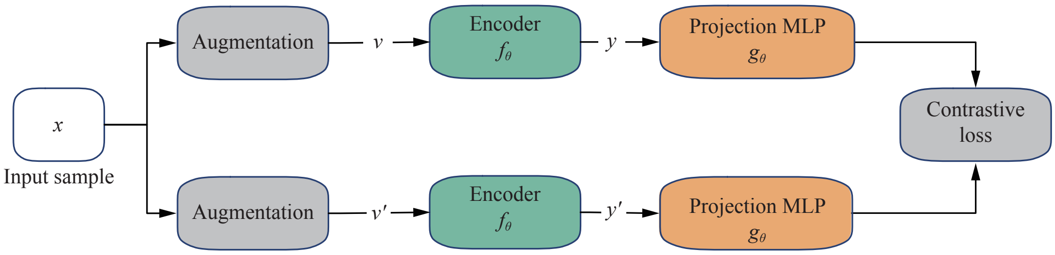

Fig. 7 Illustration of SimCLR

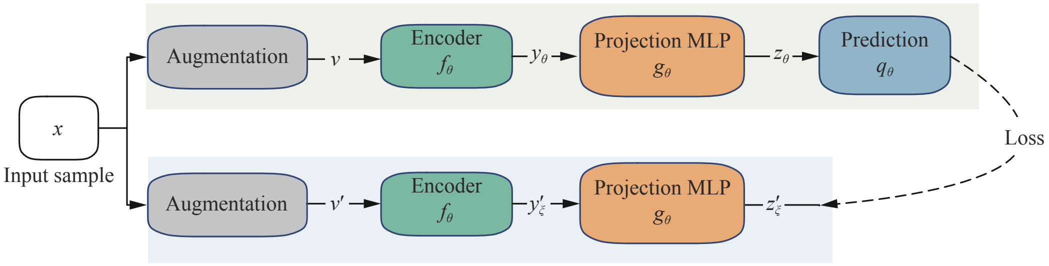

Fig. 8 Illustration of BYOL

tion from the predictor of the online network and the output of the target network projection:

$$\mathcal{L}_{\theta,\xi} \triangleq \|\bar{q_\theta}(z_\theta) - \bar{z}'_\xi\|_2^2 = 2 - 2\frac{\langle q_\theta(z_\theta), z'_\xi\rangle}{\|q_\theta(z_\theta)\|_2 \|z'_\xi\|_2} \tag{8}$$

where the $z_\theta$ is a representation ($y_\theta = f_\theta(v)$, where $v$ is the augmented sample, and $f$ denotes the encoder) passing through the projection, $z_\theta = g_\theta(y_\theta)$; $q_\theta(z_\theta)$ is the prediction in the online network; $z'_\xi$ is the projection of the target network; and $\|\cdot\|_2$ denotes as the $l_2$-normalization. When the parameters $\theta$, $\xi$ are updated, the stochastic optimization step will minimize the total loss of BYOL, $\mathcal{L}^{BYOL}_{\theta,\xi} = \mathcal{L}_{\theta,\xi} + \widetilde{\mathcal{L}}_{\theta,\xi}$ where $\widetilde{\mathcal{L}}_{\theta,\xi}$ represents the augmented sample $v$ feeding into the target network while the other augmented sample $v'$ enters the online network.

Simple Siamese networks (SimSiam)[122] proposed a hypothesis on the implication of the stop-gradient, which plays an indispensable role in preventing collapsing effectively, as shown in Fig. 9. Practically, it is the same as SimCLR, BYOL, SwAV, etc., which all utilise the Siamese network, that a single sample is augmented to two views and then processed by the same encoder network. The difference is that on one side, the encoder is followed by a predicted MLP, while the other side has no MLP but with a stop-gradient operation. The model is optimized by maximizing the similarity between the outputs of the predictor and the encoder. Concretely, we can formulate the process to the outputs of $p_1 = h(f_\theta(A(x_1)))$ and $z_2 = f_\theta(A(x_2))$; minimizing their negative cosine similarity with the following equation:

$$\mathcal{D}(p_1, z_2) = -\frac{p_1}{\|p_1\|_2}\frac{z_2}{\|z_2\|_2} \tag{9}$$

where $\|\cdot\|$ represents the $l_2$-norm. The final loss can be defined as a symmetrical loss with a stop-gradient operation ($sg$) as

$$\mathcal{L} = \frac{1}{2}\mathcal{D}(p_1, sg(z_2)) + \frac{1}{2}\mathcal{D}(p_2, sg(z_1)) \tag{10}$$

where $p_2 = h(f(A(x_2)))$ and $z_1 = f(A(x_1))$.

Masked autoencoders (MAE) are self-supervised learners that randomly mask patches of images and predict the missing pixels[121]. The architecture of MAE is shown in Fig. 10. MAE employs the random masking strategy. Specifically, MAE randomly samples and masks a portion of patches from the images based on a uniform distribution. Each masked patch is replaced by a token, a shared and learnable vector. In the MAE, the encoder is a ViT model that is only used to process visible patches to

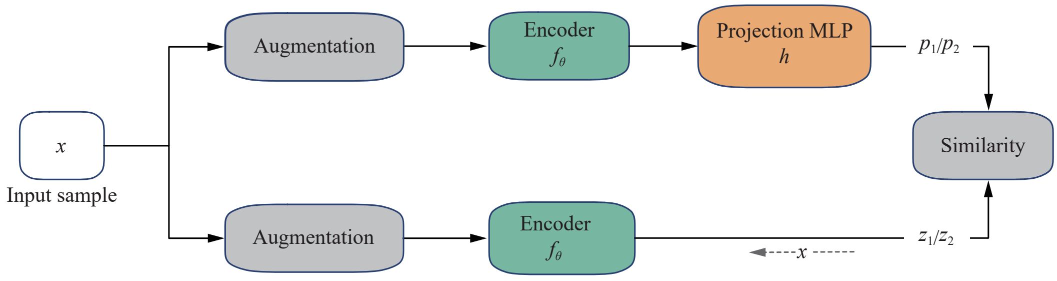

Fig. 9 Illustration of SimSiam

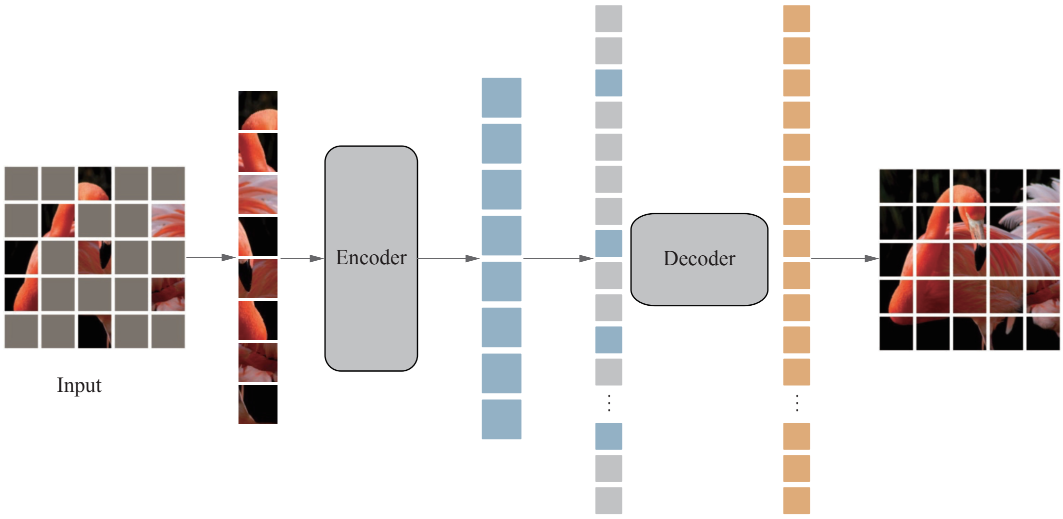

Fig. 10 Illustration of MAE architecture[121]

obtain its embeddings; the decoder is a light model built with several transformer blocks, and the last layer of the decoder is an MLP. The dimension of the MLP module's output is the same as the patches, which is used to predict the pixels of the masked patches. The encoder inputs are masked tokens and encoded visible patches combined with positional embeddings. Finally, a simple MSE loss will be introduced to calculate the value of the loss between the predicted and original pixels.

BERT[8] stands for bidirectional encoder representations from Transformers, the illustration shown in Fig. 11. Based on the investigation, BERT profoundly affects the processing and generation of EHRs data and textual medical data in the medical field[126]. The BERT model adopts the main structure of the bidirectional deep Transformer, which will be introduced next.

The Transformer is an attention mechanism that uses the structure of the encoder and decoder to calculate the relationship between input information[127]. After the input is passed through the encoder, the contribution of an input element to the total input can be calculated[127]. In natural language processing (NLP), this attention score is used as the weight of other words for that word to compute a weighted representation of a given word[128]. The influence representation of a given word can be obtained by feeding a weighted average of all word representations into a fully connected network. When passing through the decoder, only a one-word representation can be decoded in one direction at a time, and each decoding step will consider the previous decoding results[127]. After the birth of Transformer, the development of large-scale self-supervised language generation in the field of NLP is improved remarkably.

After pre-training, BERT can obtain robust parameters for downstream tasks. By modifying inputs and outputs with data from downstream tasks, BERT can be fine-tuned for any NLP task[128]. BERT can handle these applications efficiently by inputting a single sentence or sentence pair. For input, its pattern is two sentences connected by a particular token [SEP], which can represent[128]:

1) Sentence pairs in paraphrasing;
2) Hypothesis-premise pairs in implication;
3) Questions in question answering-paragraph pairs;
4) Single sentences for text classification or sequence tagging.

For output, BERT will generate a token-level representation for each token, which can be used to process sequence labelling or question answering, and unique tokens [CLS] can be fed into an additional layer for classification[128].

In this section, we provide a high-level introduction to benchmark datasets in the medical domain and representative pre-training strategies, as this paper focuses on pre-training in the medical domain, which will make the readers who are not specialised in pre-training techniques quickly and clearly learn about the developments of the related methods and the latest techniques.

## 3 Medical images in pre-training

The CV technique has been widely used in medical imaging, providing excellent technical support for clinical tasks[129]. In the fold of medical imaging, three main tracks are receiving more attention, such as diagnosis, segmentation, and survival prediction. The image data modalities include CT[130], MRI[131], X-ray[132], Ultrasound[133], Dermoscopy[55], Ophthalmology[134], whole slide tissue images (WSI)[60], etc. In recent years, learning in medical images has changed from traditional heuristic learning to learning-based learning, which means that new learning methods can obtain essential information from a large number of unlabelled medical images[135]. The information in medical pictures can either be marked manually or extracted by the mechanism of a deep learning network. The manual annotation of datasets with over millions of samples is very expensive, and the privacy of medical information is also essential because the information of many medical image data is not shared, especially some particular disease images[136]. In terms of that, the concept of transfer learning has been considered

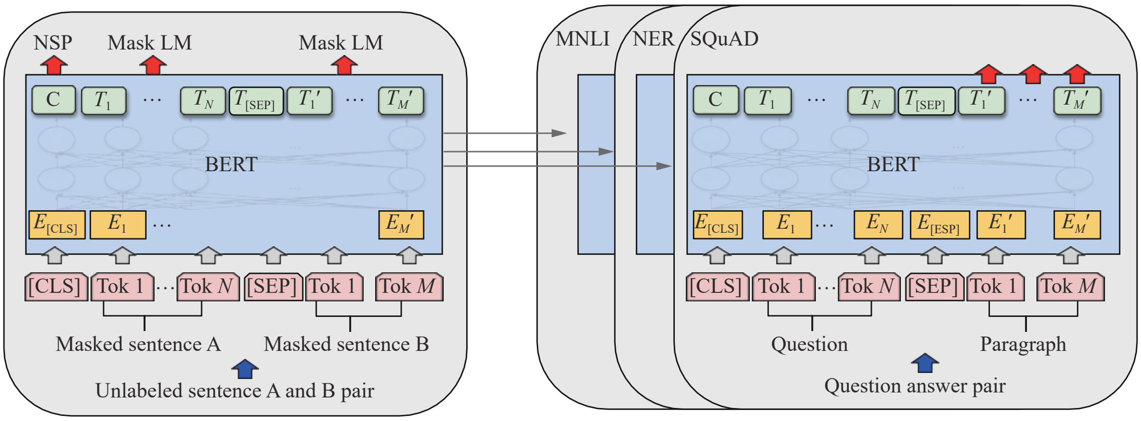

Fig. 11 Illustration of BERT[8]

with a smaller number of labelled images. Some ImageNet pre-trained models are used in processing medical imaging tasks. However, in practice, ImageNet pre-trained models are not compatible with the downstream task of medical images, so self-supervised learning is also developing rapidly in the field of medical images[95]. Theoretically, a pre-trained model used in medical tasks not only significantly reduces the labour cost of data processing but also improves the efficiency of the model learning process. The investigation of current pre-trained models in medical images will be classified by diverse medical tasks.

## 3.1 Diagnosis

In discussing pre-training of medical images for the diagnosis, the classification characteristics of brain tumours are mainly investigated because of the large number of studies on this type of data set.

### 3.1.1 CT/MRI

Early diagnosis is crucial for treating brain tumours; however, separating the MR effects of the brain into the tumour and normal processes is a time-consuming task. Reference [137] is an early transfer learning method using pre-trained brain tumour recognition, using the large natural image dataset ImageNet, and achieved 81% accuracy in the leave one out cross-validation method. Reference [130] is an early article on extracting lung tumour data features through CT slice pre-training. It investigates using pre-training methods to improve models that predict long-term and short-term survival probability. Prakash and Kumari[138] proposed a method for transfer learning. Using VGG16 and ResNet pre-trained on ImageNet, combined with magnetic resonance brain images from the Harvard Medical School database, the final result can reach 100% classification accuracy. Compared with the previous classification results of ordinary CNN, the pre-training of transfer learning combined with the downstream task technology of image enhancement improves the classification accuracy. Khan et al.[139] proposed a data-augmented pre-training method, compared to previous VGG16 and ResNet, the designed CNN model is applied on a small MRI brain slice dataset for brain tumour detection, and this method is faster and more accurate. Based on the previous augmented data treatment, Sajjad et al.[140] used a CNN model to segment the tumour region, used the data augmentation mode to expand the segmented data for pre-training and then fine-tunes the pre-trained CNN model. The article uses many brain tumour data sets to compare the model results. Compared with the tumour grading results before data augmentation, the transfer learning pre-trained model after data augmentation has higher accuracy.

Deepak et al.[141, 142] extract the features of brain tumours in the data set in the pre-training of transfer learning and simulate the size of different pre-training data in the experiment. The data set is taken from [143] on brain tumours. In [141], the authors employed Support Vector Machine (SVM) and KNN classifiers in the downstream tasks. The results indicate the exceptional capability of the pre-trained model to extract valuable features from brain MRI images. The integration of the pre-trained model enhances robustness and yields superior performance with a small amount of training data. The data set will have relatively good results after testing, but the recognition of brain tumours type meningioma is not as good as the other two.

Small-scale tumour image datasets are common, so there are some investigations on the pre-trained methods to mitigate the influences of the insufficient scale of image data. Swati et al.[144] used transfer learning to perform brain tumour classification studies on brain magnetic resonance images, pre-training on small-scale data, and the dataset is [145]. The experimental results on this dataset are state-of-the-art on a small scale of data. Wang et al.[146] discussed the use of ResNet to pre-train on the public Luna16 dataset and then fine-tune it on the lung cancer data of Shandong Provincial Hospital, with an accuracy rate of 85.71%, which is better than the existing AlexNet, VGG16 and DenseNet on lung cancer. Also, in small (100 sample lung CT images) training, a high level of performance can still be maintained.

Moreover, many novel pre-trained structures in medical image diagnosis appeared with reliable performance. Marentakis et al.[147] investigated the classification of CT images of non-small cell lung cancer (including adenocarcinoma and squamous cell carcinoma) and compares it to four types of technical models. It is found that the structure of long short-term memory (LSTM) + Inception performs the best, which is better than experts' classification accuracy, which is 7%–25% higher. Because of LSTM, this model is not allowed to perform segmentation on the image, so the technique is not affected by differences in the edges of the image. Kutlu and Avcı[148] established a new model to classify liver and brain tumours. First, the pre-trained model of the AlexNet architecture is used to extract features from the input data, and then the features of wavelet transform can be used to extract essential factors to improve the classifier's performance. Finally, LSTM has the function of the signal classification.

### 3.1.2 Ultrasound

Given that current radiologists' professional skills and knowledge are not very reliable, many abnormal ultrasound images of fatty liver cannot be well diagnosed, leading to the development of fatty liver into a fatal chronic disease. In order to improve the accuracy of ultrasound image classification, Reddy et al.[133, 149] proposed the convolutional neural network combined with transfer learning (VGG16 pre-train) to analyse and identify whether there is fatty liver. At the same time, these two articles compare the ordinary CNN without pre-training and other non-deep learning methods. The results show that the pre-training and fine-tuning of transfer learning have significantly improved the recognition rate, both of which remain above 95%.

#### 3.1.3 X-ray

Benign and malignant breast tumours are difficult to distinguish under X-rays. In the recent popular transfer learning, most pre-trained models are trained on the mainstream ImageNet benchmark datasets. Since these datasets do not contain breast-related images, the recognition results are not ideal. Alkhaleefah et al.[150] proposed a double-shot transfer learning model. The pre-training uses the image enhancement mode to reduce the problem of over-fitting and insufficient data. Compared with other mainstream pre-training models, the recognition accuracy is greatly improved. In [132], two models are designed to improve the recognition of lung diseases by X-ray and CT, respectively: one is an improved AlexNet, and the other is a combination of human operation and pre-trained learned features to improve the classification accuracy. The improved pre-training method improves the accuracy by about 10% compared to the original training method. The two training datasets are [151] and [152].

### 3.2 Segmentation

#### 3.2.1 Abdominal organ segmentation

The abdomen is a vital part of the area in the human body, referring to the region between the thorax and pelvis[27]. Abdominal organ segmentation has significance for reducing patients′ mortality. In the task of abdominal organ segmentation, single or multiple abdominal organs are segmented into semantic segments of pixels identified with homogeneous features, such as colour and texture[27]. Automatic segmentation of lesions on liver images is an essential step for correct decision-making in clinical diagnosis. In [153], a cascade fully convolutional neural network is proposed to automatically segment the liver and lesions in CT and MRI abdominal images. The first convolutional network was used to identify the location of the liver in the abdominal picture, and the second network was used to identify the lesion site. Both convolutional neural networks are pre-trained using U-Net[154]. The accuracy of the experimental results of Dice has reached the current best level, and the model can be fine-tuned to adapt to different situations. Conze et al.[155] designd a multi-organ segmenter for CT and MRI images of the abdomen and extends standard conditional generative adversarial networks. At the same time, Cascade′s pre-trained encoder-decoder structure extracts the features of organs and identifies abdominal organs through contextual information. The adversarial generative network in the paper achieves better segmentation than the encoder-decoder structure. In addition, Kavur et al.[44] introduced a medical image segmentation competition held at CHAOS dataset, in which players use the prestained encoder-decoder model for training for single or multiple organ segmentation tasks. In unimodal and multi-modal tasks, pre-trained deep learning models show comparative advantage results compared to other methods.

The training of 3D images is not efficient. Therefore, Li et al.[48] proposed a method using a 2D UNet (H-DenseUNet) and a corresponding 3D DenseUNet for liver cancer segmentation computation. The convergence speed of the pre-trained transfer learning is significantly higher than that of the ordinary model. This method was evaluated on the MICCAI 2017 liver tumour challenge[47] and the 3DIRCADb dataset[156], respectively, and also achieved state-of-the-art results. The application of 2D and 3D image models has merged algorithms with time advantage and memory space advantage.

#### 3.2.2 Ultrasound

Convolutional neural networks have shown promising results in breast tumour segmentation in ultrasound. Generally, these CNN-based methods modify the architecture model or use the CNN ensemble to design new models. Gómez-Flores and Pereira[157] evaluate the segmentation of breast tumour ultrasound images using four transfer learning models, including AlexNet, U-Net, VGG16 and VGG19′s SegNet, and ResNet representations. These pre-trained models are fine-tuned on normal and tumour breast images, where the datasets come from [50, 158]. In these ultrasound breast-specific datasets, the F1 value of the test after pre-training on ResNet18 is the highest, which indicates it has more potential capability. Similarly, in [159−161], investigations of the segmentation of ultrasound breast cancer with transfer learning are included, and [162] also made the comparison of kidney image segmentation with pre-trained methods. In contrast, these investigations prove that pre-training can be efficiently and precisely used for ultrasound image feature extraction.

#### 3.2.3 Comprehensive

The application of self-supervised learning in segmentation is also pervasive. Based on many basic computer vision models, especially the method of exchanging segmentation positions, the pre-trained encoder can accurately learn the features of the picture. Bai et al.[163] used U-Net to pre-train and test the accuracy of the training set of tiny hearts, and the experimental result improves the accuracy by about 0.04 compared with the ordinary U-Net. Li et al.[164] conducted a pre-training experiment on image rotation, which is also a popular model for self-supervised learning. The method is similar to SimCLR′s image enhancement, derived from the model relative positions of image patches[165, 166]. This method performs pseudo-label classification for clusters in the results of self-supervised learning. Experiments show that this pseudo-label pre-training method reduces labour costs by 80% and achieves the same level of segmentation accuracy. The pre-training of self-supervised learning mainly does not have annotated requirements for the input information, and the data characteristics of the pictures learned under the encoders of different pre-training methods are more similar to the intrinsic data features. Chen et al.[167] proposed a method to learn semantic features of medical images using self-supervised learning while investigating the effect of unlabelled pre-training applications for classification, localization, and segmentation. Semantic features can be appropriately learned by the context restoration method, and the results of various pre-train scenarios prove that self-supervised learning has reliable performance on medical image tasks.

## 3.3 Survival prediction

As well as classification and segmentation tasks have received much attention, survival analysis also plays a critical role in current clinical practice as a part of computer-aided medical image analysis. Survival prediction (survival analysis or prognosis) is a medical task to predict the expected duration of time until events happen (e.g., death), which is frequently used for cancer patients[60]. Some works have utilised deep learning methods to achieve state-of-the-art survival prediction results[168, 169]. However, the requirement for large amounts of well-phenotyped training data has still been one of the significant challenges for introducing deep learning into survival prediction[170]. There are very few large, labelled, and public datasets. It may be possible to overcome the challenge of limited data by pre-training on a large dataset from another domain[170]. Therefore, some works introduce pre-trained models or pre-trained strategies. Li et al.[171] considered in the survival prediction that the interesting event may not be observed during the study period, and collecting sufficient annotated training samples for robust prediction is extremely difficult in real practice. A transfer learning-based Cox method, namely Transfer-Cox, was proposed to use auxiliary data in a situation where the training data is insufficient. This method aims to extract valuable knowledge from the source domain and transfer it to the target domain with the $L_{1,2}$-norm penalty for learning a shared representation across the source and target domain. Agravat and Raval[172] demonstrated a CNN architecture for glioma segmentation and feature extraction, and the extracted features are used to predict the survival of patients with random forest regression. To reduce the impact of high imbalance in the brain tumour segmentation task, in the initial stage, the network is trained for the whole tumour, which provides tumour localization in the brain, and in the next stage, the parameter of the network in the first stage will transfer to process sub-component (e.g., oedema, enhancing tumour and necrotic core). Yao et al.[60] developed the deep attention multiple instance survival learning (DeepAttnMISL) model to predict cancer survival accurately. For the feature extraction process, they used an ImageNet pre-trained VggNet to extract features from image patches, and Setio et al.[173] found that using medical pre-trained models positively impacts survival tests for two survival prediction approaches, DeepAttnMISL and WSISA[169]. Chen et al.[174] proposed the hierarchical image pyramid transformer (HIPT), two stages self-supervised pre-training framework to leverage the natural hierarchical structure inherent in WSIs to learn high-resolution image representation for cancer sub-typing and survival prediction. The self-supervision part of this work uses student-teacher knowledge distillation (DINO), where one of the paths in Siamese is the teacher network and another is the student network.

## 3.4 Longitudinal images data

The images captured from the same area at different times can be considered time-series data. For instance, the CT images of the same body area scan at 1, 3 and 6 months, respectively[175]. The set of images can be regarded as time-series images data that contain abundant temporal relevant diagnostic information. Integrating temporal information into medical imaging learning has significance for enhancing the diagnosis, prognosis, and disease progression analysis[175–177]. Some previous works used the CNN and recurrent neural network to mine the temporal and spatial information simultaneously[176, 178]. However, with our investigation, only a few works are involved in employing a pre-training approach in this field. Xu et al.[175] demonstrated using the deep learning method to predict prognostic endpoints of patients treated with radiation on longitudinal CT imaging obtained follow-up. In this work, they use ImageNet pre-trained model to extract CT image features. Ouyang et al.[179] proposed a longitudinal neighborhood embedding (LNE) to capture the gradual deterioration of brain structure and function caused by ageing. They construct a smooth trajectory field that is built by graph construction in each training iteration in the latent space to capture the global morphology while maintaining the local continuity. The extensive experiments demonstrate that the LNE is positive for exploiting the association of information temporal and spatial to reveal the impact of neurodegenerative disorders. Ren et al.[131] presented a local and multi-scale spatial-temporal representation learning method for pre-training on longitudinal MRI imaging datasets, while they proposed various regularisations for avoiding collapsing when extending to multi-scale spatial-temporal representations. They evaluated the improvement in longitudinal neurodegenerative adult MRI and developing infant's brain MRI for segmentation tasks. Konwer et al.[177] proposed a framework to improve clinical prediction tasks using limited temporal medical images. The proposed framework consists of two modules: temporal progression learning and snapshot learning. The temporal progression learning extracts temporal image sequences using a temporal ConvNet and a self-attention module. Snapshot learning includes self-supervised learning on unlabelled data and then using the target data to fine-tune the network. A re-calibration network is utilised to align these two contextual representations. The experiments demonstrate that this framework outperforms other advanced clinical prediction methods.

### 3.5 Section conclusion

In all, the main progress of medical images comes from the new field proposed by computer vision, and the impact of pre-training on traditional machine learning and deep learning is huge. Table 3 illustrates the major papers discussed in this section. Transfer learning and self-supervised learning solve the problem of image labelling and the problem of fewer data in pre-training, and the accuracy of pre-training segmentation and diagnosis can generally achieve more accurate results than traditional supervised learning. The application of pre-training on pictures greatly improves the function of auxiliary medical detection, reduces the workload of doctors, and improves the reliability of diagnosis.

Table 3 Summary of pre-training related publications with diverse image types in the medical field

| Tasks | Image type | Papers |
|---|---|---|
| Diagnose (Classify) | CT/MR | [130, 137–142, 144, 146–148] |
| | X-ray | [133, 149] |
| | Ultrasound | [132, 150] |
| Segmentation | Abdnomial | [44, 48, 153, 155] |
| | Ultrasound | [157, 159–161] |
| | Comprehensive | [163, 164, 167] |
| Survival prediction | WSI | [58, 60, 171, 172, 174] |

## 4 Bio-signal data in pre-training

It is known that there are several different types of bio-signal data in the medical domain, such as electroencephalograms (EEG), electrocardiograms (ECG), heart rate variability (HRV), electromyograms (EMG), electrodermal activity (EDA) and photoplethysmography (PPG), of which contain a large volume of physiological information. DL-based advances in bio-signals enable the processing of signals (signal segmentation, wave detection, and noise removal) and the creation of high-quality feature representations to be used in clinical applications, such as signal de-noising, disease diagnosis, emotion recognition, genetic mutation detection, etc. EEG and ECG are representative bio-signal data, and there are many publicly available datasets, most of which are generally annotated. Therefore, we collect many pre-trained related papers based on EEG and ECG signals, not to say that the other medical time-series data is unimportant.

Despite a massive breakthrough in algorithms and the increasing availability of publicly available datasets, the lack of annotated data continues to pose one of the most significant challenges to developing bio-signal processing in artificial intelligence. Some researchers have been using pre-training model techniques on many different tasks to address the data scarcity issue. In the remainder of this section, we summarise the current state-of-the-art research on using pre-training methods to process bio-signal datasets based on tasks of all kinds based on bio-signal datasets.

### 4.1 Pre-processing

Pre-processing the raw signals is one of the essential steps for bio-signal-related research. The raw data contain multiple noises that are probably caused by different factors. Antczak[180, 181] proposed an approach that uses the pre-trained model to remove the noise from the raw ECG data; notably, they pre-trained the model with the synthetic data and fine-tuned the real data to create a state-of-the-art noise-removing neural network. In addition, QRS wave detection is an essential task for ECG prepossessing. Rodrigues and Couto[182] utilised transfer learning to detect the QRS wave and predict the next QRS wave and the shape of the next ECG segment.

### 4.2 Disease diagnosis

Disease diagnosis with bio-signal data has a vast range of applications since it has been well-studied in various scenarios. Specifically, tasks include arrhythmia diagnosis, atrial fibrillation diagnosis, epileptic seizures detection, etc. Pathak et al.[166, 183] attempted to use the pre-training method to develop an automatic arrhythmia diagnosis system on one dataset and fine-tuning it on another dataset to evaluate the effectiveness of the pre-training model for ECG data, in which the data used in the tasks were all labelled by cardiovascular experts and the dataset used for pre-training and the fine-tuning under the same tasks. The works[16, 184, 185] employed the same method to detect atrial fibrillation (AF), but they trained the model on a general ECG dataset and fine-tuned it on an AF dataset. In addition, this type of transfer learning can be applied to EEG datasets to diagnose epileptic seizures in a similar way[200, 201], which all utilise the CNN network, and Raghu et al.[200] converted the EEG signals into images using short-time Fourier transforms (STFT), while Nogay and Adeli[201] processed the raw EEG with 1D-CNN. However, the data are usually not annotated in the real world; therefore, Weimann and Conrad[16] evaluated the performance of the unsupervised pre-training model, and, as reported in the study, unsupervised or self-supervised pre-training yielded a lower performance than supervised pre-training, but they will become more relevant because they rely on fewer annotations.

To leverage the unlabelled bio-signals, like [16], Thinsungnoen et al.[35] designed auto-encoder networks training bio-signal representations and then clustering features. In recent years, the emerging family of self-supervised (contrastive) learning methods has been applied to bio-signals data[186–192, 199, 202–204]. Mehari and Strodtholt[188] assessed self-supervised representation learning from 12-lead ECG data using SimCLR, BYOL, and CPC, from which CPC got the best results that only fell behind 0.5% supervised performance. Liu et al.[189] also explored using the self-supervised learning approaches to detect arrhythmia; unlike [188], they converted ECG signals into grey-scale bitmap; additionally, they emphasised that the self-supervised learning can alleviate the problem of label imbalance and significantly reduce the quantity of requirement for annotated data. For EEG data, Mohsenvand et al.[199] presented sequential contrastive learning of representation (SeqSLR) to diagnose epilepsy, which is based on the channel-wise feature extractor based on SimCLR, demonstrating that it outperforms conventional contrastive learning frameworks. A self-supervised pre-training framework, contrastive learning of cardiac signs (CLOCS), specifically designed for cardiac signals, is used to exploit the cardiac data across space, time, and patients[186]. Zhang et al.[191] proposed a general bio-signal framework referred to as time-frequency consistency (TF-C) by contrasting the samples in the time domain and the frequency domain, evaluating it in diagnosing the arrhythmia using ECG, epilepsy using EEG, and muscular diseases using EMG data. The latest work from Tang et al.[202] proposed a self-supervised graph neural network to diagnose seizures on EEG, demonstrating that the self-supervised pre-training has consistently improved. It represents the spatial-temporal dependencies in EEGs using GNN and the self-supervised pre-training strategy to improve performance.

## 4.3 Emotion detection

Emotion detection has become an emerging field of study in computer-aided learning to equip machines with the ability to recognise the emotional states of individuals[210]. An emotion can be considered a physiological and psychological expression that can be detected by many types of bio-signals, such as electrocardiograms (ECG), electrooculograms (EOG), galvanic skin responses (GSR), etc.[195] The emotion computation analysis with DL and ML has achieved success, like stress or anxiety level detection[194, 211], personality analysis[212], emotion recognition[193], etc. However, aside from the lack of data and annotations, emotion detection systems expect generalised models that can take into account the state of the emotion from a global perspective, and these generalised models can transfer to other tasks. Taking a multi-task generalised model, for example, can be transferred to a specific emotion task[195], where the process of training this generalised model takes into account the pre-training techniques. To investigate if the pre-trained models enable enhanced performance in emotion detection, Radhika et al.[193, 197, 198] pre-trained CNNs with annotated data and fine-tuning on target source data. Among them, Cimtay and Ekmekcioglu[197] stated that they applied the pre-trained model to cross-subject and cross-dataset EEG signals and reported promising results. In our survey, we have found that some recent emotion detection tasks learn the generalised pre-training feature representation through self-supervised learning methods. For instance, Sarkar and Etemad[195] proposed a self-supervised network to pre-train the feature embeddings on an aggregation of four publicly available datasets to overcome the challenge of having different types of output labels for each dataset. In comparison to training on individual datasets, the framework with a pre-trained model performed better in emotion recognition. Furthermore, they proposed a self-supervised representation learning framework to detect maternal and fetal stress on ECG data and applied it in real-world practice[194]. From EEG data, Mohsenvand et al.[199] evaluated their proposed SecCLR framework in emotion recognition on the SEED dataset.

## 4.4 Sleep stage detection and other tasks

Sleep stage detection aims to determine the sleep stage from polysomnography (PSG), EEG, EOG, and EMG. Phan et al.[207] proposed SeqSleepNet+ and DeepSleepNet+ frameworks with pre-trained models to classify sleep stages. They conducted pre-training on one type of signal and fine-tuning on another type. For example, they pre-trained the model on ECG and EOG source set and fine-tuned it on EEG and EOG target set. Pre-trained SeqSleepNet+ and DeepSleepNet+ models resulted in a significant improvement in sleep staging performance. Banville et al.[204] investigated and explored using self-supervised learning to pre-train feature representations on EEG-based sleep staging detection. Jiang et al.[208] proposed a self-supervised contrastive pre-training method to conduct representation learning of EEG signals applied to sleep stage tasks. With more unlabelled data available for the network, the proposed method reached 88.16% accuracy on the Sleep-EDF dataset. The model TF-C[191] evaluated the performance on the sleep staging classification task.

Other bio-signal data tasks also employ the pre-training model approach to learn their feature representation. Aston et al.[196] extracted features from the two-dimensional attractor generated from the ECG signal by the novel symmetric projection attractor reconstruction (SPAR) method used to detect a mouse's genetic mutation using pre-trained models that were trained on the ImageNet dataset. Identifying motor and mental imagery is a vital topic in brain-computer interface (BCI) research that recognises the subject's intention to such as implement prosthesis control[213]. Amin et al.[5] and

Sadiq et al.[205] utilised the fully-supervised pre-trained models to enhance the performance on the small EEG BCI datasets. Cheng et al.[190], and Jiang et. al.[208] proposed the self-supervised learning-based model to overcome challenge of performance degradation under a small number of labelled training samples.

### 4.5 Section conclusion

This section summarizes recent studies that pre-train feature representations and use the pre-trained model on downstream tasks on bio-signal data. Table 4 lists the related tasks and the corresponding citations. These studies have all shown the pre-training techniques to succeed in specific scenarios. However, many limitations exist in current studies. First, some studies have shown that pre-training does not lead to any notable improvements in the tasks. However, it can significantly reduce the training time and speed up the convergence process. Additionally, no large-scale dataset supports pre-training a generalized and high-quality representation of features. Therefore, for bio-signals, a specific pre-training framework is required to explore to get further improvements in the performance, such as CLOCS[186], SeqSLR[199], and TF-C[191]. Furthermore, although Liu et al.[189] showed that self-supervised learning can alleviate the class imbalance problem, the class imbalance has remained a standard issue for the bio-signal dataset, yet only a few works have attempted to address the issue.

## 5 EHRs in pre-training

In comparison with pre-training in other areas, there are fewer exploration opportunities for EHR data. In this section, we summarize the latest research for EHRs based on pre-training. As listed in Table 5, this table presents a compilation of various tasks related to Electronic Health Records (EHRs) along with relevant studies conducted in each of the tasks. Pre-training has been extremely successful in many areas. EHRs data-related tasks are one of the areas where pre-training has had a significant impact. In conditions where there is a lack of data, it is possible to enhance the performance of the model[214]. The EHRs-related tasks include prediction[33, 126, 214–222], information extraction from clinic notes[223–226], the international classification of disease (ICD) coding[227, 228], medication recommendation[229, 230], etc.

### 5.1 Prediction

AI-aided prediction is critical in clinical practice, automatically analysing patients' conditions and providing suggestions to doctors for saving more lives. The primary purpose of prediction in EHRs is to predict the progression of the disease, such as mortality, the next visit, etc. For example, Rasmy et al.[126] utilised the pre-trained model referred to as Med-BERT to predict heart failure among patients with diabetes and the onset of pancreatic cancer on the Truven and Cerner EHR datasets. They developed a domain-specific cross-visit pre-training model based on the BERT model. Med-BERT achieved promising performance on disease prediction tasks with small fine-tuning datasets and enabled to boost the AUC by more than 20%. However, if the pre-trained model consists of abundant auxiliary tasks and has a complex relationship to the target task, using the pre-trained model becomes inefficient and subnormal[217]. Xue et al.[217] proposed a method to automatically select from a large set of auxiliary tasks to address the challenge. They employed the self-supervised pre-training and the pre-trained model to predict clinical outcomes. Tipirneni and Reddy[218] proposed a self-supervised transformer for the time-series model (STraTS) to predict clinical outcomes, which overcomes the challenges of sparsity and irregular time intervals in EHRs-related works; meanwhile, STraTS leverage unlabelled data for tackling the issue of limited availability of labelled data. McDermott et al.[214] established a pre-training benchmark dataset for EHR time-series data to which various fine-tuning tasks are conducted, filling an essential hole and providing a baseline for pre-train-

Table 4 Summary of bio-signal data in the medical domain based on pre-training. PT w labels: pre-training with labels; PT wt labels: pre-training without labels; Semi PT: pre-training using semi-supervised learning.

| Datasets | Tasks | PT w labels | PT wt labels | Semi PT |
|---|---|---|---|---|
| ECG | De-noise | [180] | [181] | |
| | QRS detection | | | [182] |
| | Diagnosis/Classification | [15, 16, 107, 183–185] | [16, 35, 186–192] | |
| | Emotion detection | [193] | [194, 195] | |
| | Detection of genetic | [196] | | |
| | Emotion detection | [197, 198] | [199] | |
| EEG | Disease detection | [200, 201] | [191, 199, 202–204] | |
| | Identify motor mental imaginary | [5, 205] | [190, 206] | |
| | Sleep stage detection | [207] | [191, 204, 208] | |
| | Multi-task | | [209] | |

Table 5 Summary of EHRs-related tasks based on pre-training

| Tasks | Related papers |
| --- | --- |
| Prediction | [33, 126, 214–222, 231, 232] |
| Information extraction | [223–226] |
| ICD coding | [227, 228] |
| Medication recommendation | [229, 230] |

ing on EHR data. They evaluated the benchmarking with self-supervised pre-training and weakly-supervised multi-task. Xu et al.[216] introduced the medical knowledge graph combined with self-supervised pre-training to deal with the sparsity and high-dimensional issue of EHR data. Lu et al.[221] utilised a pre-trained model to detect disease complications and compute the contributions of particular diseases and admissions. Using the self-supervised learning method, the pre-trained model was trained based on the hidden disease representation. Meng et al.[33] proposed a model that can process five heterogeneous and high-dimensional datasets in a temporal manner in order to predict chronic diseases, such as depression. Aken et al.[220] conducted clinical outcome pre-training to integrate knowledge about patient outcomes from multiple public sources. The model learns a representation for clinical outcomes, in which the model learns a relation between symptoms, risk factors, and clinical outcomes. Chen et al.[215] proposed the physiological signal embeddings (PHASE) framework to forecast adverse surgical outcomes accurately. PHASE is a self-supervised-based model that learns the representations of the physiological signal and then uses the other prediction method to forecast the outcome. In addition, considering privacy issues, they attempted to simulate transferring the pre-trained model between organisations. The conventional sequential models are difficult to reuse for the early diagnosis of pregnancy complications; therefore, Ren et al.[219] proposed the representation by pre-training time-aware transformer, particularly for the early diagnosis of pregnancy complications. In this task, they designed three pre-training tasks to handle data insufficiency, incompleteness, and short sequence problems. Hur et al.[222] designed description-based embedding (December), a code-agnostic description-based representation learning framework for predictive tasks. They evaluated the performance of the proposed model on prediction tasks, transfer learning, and pooled learning. No uniform standard in EHRs is limited to applying the trained prediction models well to other EHR datasets from different organizations. To this end, Sun et al.[231] proposed a generic transfer learning strategy that first pre-trains the model on source datasets then transfers the best-performing pre-trained model to target datasets for fine-tuning the network. Ma et al.[232] proposed a distil transfer learning framework, DistCare, for prognosis. DistCare leverages the existing EHR data, thus reducing the impact of the available data limited to the rarity of cases and privacy issues. Specifically, they pre-trained models on the publicly available COVID-19-related EHRs, regarded as the teacher model based on distillation to obtain more comprehensive representations of source datasets. A series of extensive experiments on different clinical tasks and datasets show that DisCare benefits the prognosis with limited data.

## 5.2 Information extraction

EHR data contains valuable information which can assist doctors in diagnosing and making the treatment scheme. Recent studies have introduced pre-training in processing EHR data. For example, Chen et al.[224] utilised the BERT pre-training EHR data embeddings to extract features with the structural data. The extracted features are then used to train with SimCLR and Deep InfoMax (DIM) under an unsupervised learning strategy to embed the disease concept. The pre-trained model further fine-tunes to adapt to the target outcome prediction task. Zhang et al.[226] proposed the DeepEnroll model, which combines enrollment criteria and patient records into a shared latent space using a cross-modal inference learning approach. DeepEnroll encodes the patient's EHR data using the pre-trained BERT model. A real-world dataset was applied to this approach, and impressive results were achieved. Most existing studies do not consider capturing the time doctor experience and expertise with time-evolving in EHR data and learning static doctor representation. Biswal et al.[223] proposed the Doctor2Vec model, which simultaneously enables learning the doctor representation and trial representation. The model achieved an 8.1% relative improvement in PR-AUC compared with the baseline model relying on dynamic doctor representation learning and pre-training a BERT model to understand trail descriptions. As the survey shows, some of the models on EHR data utilised the transformer-based model as the pre-train model, like BERT. However, in real-world clinical practice, there is an amount of privacy and sensitive information in the EHR data. In order to investigate whether the pre-trained embedding can be converted into the original information, thus causing privacy leakage, Lehman et al.[225] executed experiments attempting to recover the personal healthcare information from the feature embeddings. They stated that a simple attack could not recover sensitive information, but more sophisticated methods could do this.

## 5.3 ICD classification

ICD coding is the task of predicting and coding all doctors' diagnoses with clinical test notes containing patients' symptoms and diagnostic procedures in an unstructured text format[233]. Recent studies have provided evidence suggesting that DL and ML can classify the ICD coding. Data annotation is a time-consuming task, while for clinical text notes, annotation requires professional expertise. To solve the lack of annotation issue, some researchers focus on using pre-training methods[227, 228]. Hlynsson et al.[227] proposed a semi-self-supervised ICD coding framework. They attempted to train pre-trained models with four existing transformer-based models for clinical feature extraction and then use the data with the label to train a logistic regression ICD classifier. Zhang et al.[228] utilised the BERT model to pre-train on a large-scale dataset. Unlike Hlynsson's work, they introduced a multi-label attention method to train the classifier.

### 5.4 Medication recommendation

Medication recommendation is a hot topic in healthcare. It aims to recommend a set of medicines according to the patient's symptoms, which would play a critical role in assisting doctors in making decisions[234]. Meanwhile, it could be a potential strategy to mitigate the doctor shortage problem in some countries. Technically, medication recommendation systems are trained on EHR data. Existing methods only utilise longitudinal EHRs with multiple visits while ignoring a large number of patients with a single visit. Shang et al.[230] proposed the graph BERT (G-BERT) for medical code representation and medication recommendations to overcome this issue. G-BERT is the first model that introduced a language model pre-training strategy to the medical domain. Considering capturing local and global dependency information from records of patients, Su et al.[229] proposed a dynamic time-aware hierarchical dependency network (TAHDNet) for medication recommendation tasks. The performance of the proposed method is superior to that of G-BERT.

### 5.5 Section conclusion

In this section, we summarised the recent advanced studies in pre-training on EHR data. These studies were based on four tasks: prediction, information extraction from EHRs, ICD coding, and medication recommendation. There is no doubt that the transformer-based model is the mainstream for EHR data pre-training-related works. Some recent studies utilised GNN as pre-training to improve performance and interpretability. The development of a privacy-related pre-training framework seems to be a promising topic in EHR studies, as discussed in [229].

## 6 Multi-modality in pre-training

Most publicly available healthcare datasets consist of multiple modalities. It is a part of nature, since the information that people are exposed to is always multi-modal. People see the colour, hear the sound, feel the texture and smell the odour. Humans leverage different senses to better understand the information they receive. A significant reason to learn from multi-modal data is the assumption that the complementary nature of the different modalities can effectively improve performance. This assumption also applies to the medical domain. For instance, to write a comprehensive clinical report, clinicians need to review the patient's medical images and assess their medical history and vital signs. Cross-modal information could potentially improve the clinicians' understanding of patients' conditions.

Advances in uni-modal representation learning provide a firm foundation for improving performance in downstream tasks. The most common modalities in uni-modal pre-training are vision and language. In 2018, the advent of BERT[128] significantly boosted representation learning in the area of NLP. Inspired by the success of uni-modal representation learning and BERT, researchers have started to look for methods to extract joint representations from multiple modalities. To date, most multi-modal pre-trained models are based on visual and textual modalities. Li et al.[235] adopted four transformer-based[127] vision-and-language (V+L) pre-trained models for medical downstream task, namely, VisualBERT[236], Uniter[237], Lxmert[238], and PixelBERT[239]. Moreover, they compared the performance of these models using AUC. According to the experimental results, Li et al.[235] demonstrated that these four pre-trained V+L models outperformed the traditional CNN+RNN approach in the radiological classification task. Furthermore, Li et al.[235] also showed the advantages of multi-modal pre-training over text-only embedding.

### 6.1 Multi-modal pre-training tasks in the medical domain

Almost all the multi-modal pre-training in the medical domain is based on V+L modalities. Therefore, most downstream tasks focus on medical images and clinical reports, such as radiology image interpretation and medical visual question answering (VQA). Radiological examination is one of the most common diagnostic procedures in medicine. Radiologists need to read a large number of radiology images daily. Introducing AI technology to generate diagnosis reports is crucial for radiology examinations, where a model is used to describe a medical image. Recent research has mainly focused on disease diagnosis and the generation of preliminary diagnosis reports. Most multi-modal studies in the medical field currently focus on this scenario. Generally, the multi-modality models in radiology are used for disease diagnosis and report generation. Wang et al.[17] introduced an image-text pre-training approach that enables learning from raw data with mixed modality data, such as images and text. Most importantly, the data come from different institutions. In specific, the core structure of this method is a transformer-based self-supervised framework for simultaneously learning chest X-rays and corresponding text reports. They evaluated their model on three real-life application tasks: disease classification, similarity search and image regeneration. Wang et al.[18] also proposed a large-scale chest X-ray dataset used to process images and corresponding reports, which provides a prominent expectation in this field. Similar to [17], Moon et al.[19] explored the representation of learning tasks in the medical domain and proposed a transformer-based pre-training architecture with the multi-model attention masking scheme, namely medical vision language learner (MedViLL) for image-text understanding (e.g., diagnosis, medical image-text retrieval, and medical visual question answering) and version-language generation tasks (radiology report generation). In the extended experiments, MedViLL demonstrated the generalization ability under the transfer learning scenario on two chest X-ray datasets. Yan and Pei[20] proposed a pre-training model, clinical-BERT, for three specific tasks, such as clinical diagnosis, masked medical subject headings (MeSH) words modelling and image-MeSH matching, through which the Clinical-BERT pre-train the model with the medical domain knowledge, rather than regarding the medical domain words and other words treated equally as MedViLL. They demonstrate that their proposed pre-training model is effective in downstream radiograph diagnosis and report generation tasks. Radiologists are always located in a small area with the most valuable information when they read medical images. In addition, many similar sentences describe generic image areas in the reports that are redundant and can be considered non-relevant noises[156]. Most works ignored these issues. To address these issues and mimic radiology experts, Li et al.[156] proposed an auxiliary signal-guided knowledge encoder-decoder (ASGK) in which they pre-train a medical language model using the medical textual information they collected.

Since annotations of medical images require the participation of experts in corresponding domains, it is hard to obtain accurate labels of large-size datasets, and the cost is high. Therefore, only a small number of existing datasets can be used in the research of VQA on medical images. Thus, inspired by the success of self-supervised pre-training methods in NLP, vision, and language space, a multi-modal medical BERT (MMBERT)[240] was proposed, which uses existing large multi-modal medical datasets to learn better image and text presentations. Compared to other state-of-the-art (SOTA) methods on VQA tasks on medical images, MMBERT achieves superior performance and provides attention maps to improve model interpretability.

## 7 Challenges and future directions

In Sections 3–6, we comprehensively reviewed and summarised the current state-of-the-art approaches using pre-training in the medical domain. For basic pre-training techniques, there are further development directions in the future study, such as improvement of computation efficiency both in the model pre-training and downstream tasks and the research for developing a none specific task models. Future research directions could be explored to maximise pre-training benefits in the medical domain. While many efforts have been devoted to this field, some challenges still need to be fully explored. In this section, we focus on discussing the challenges based on an analysis of the works mentioned above, which may stimulate a more profound study in the future. We outline several key research directions that were found when we summarised those works.

Data scarcity remains one of the most significant barriers to training a high-performance model for medical tasks. Although hospitals and other institutions can produce much healthcare data daily, those data cannot be available due to the increasingly strict data privacy clauses. Many datasets have been published for research, as we summarised in Section 2. However, the quantity of the data is still short for pre-training a general-purpose model, especially for bio-signal, EHR and multi-modality-related tasks. Therefore, it is a future direction to pre-train a general-purpose model on limited data.

Privacy concerns about healthcare data require urgent attention due to the strict data privacy clauses. Specifically, the question of whether personal healthcare information can be recovered via malicious attacks from the pre-trained feature representation has yet to be thoroughly investigated. This problem would influence whether or not pre-training techniques could be widely applied in real-world applications. Many privacy-related tasks in ML and DL have become hot topics, such as federated learning[241] and differential privacy learning[242]. It is expected that pre-training techniques combined with machine learning research relating to privacy will be a promising field for future research in the upcoming years. Many recent works have started to research this medical data privacy field, and this topic would be well worth studying.

Class imbalance is a common challenge in machine learning and deep learning. Especially in the medical domain, disease examples are always less than non-disease examples. For some rare diseases, the class imbalance issue will be extreme. If a deep learning model has been trained on a class-imbalanced dataset, the model will bias toward the majority category. Therefore, this problem is considered when we use a class-imbalanced dataset. However, we found only a few papers considering this problem in model pre-training after our investigation. Although most of the works train the model on an unlabelled dataset with unsupervised learning or self-supervised learning strategy, we could know a rough data distribution of the dataset. The imbalance issue will be considered in the training process.

In Section 6, we have introduced the multi-modality in pre-training in the medical domain. Many researchers have tried to introduce pre-training to process the multi-modality data. However, most of the current research only focuses on generating clinical reports and tries to use the model to interpret the radiological examination, and the main reason is that there are many large datasets for this task. In contrast, the lack of task-related datasets limits the progress of research on multi-modality pre-training. Furthermore, there are currently few works on applying pre-training for bio-signal data to make survival predictions. Since bio-signal alone may not provide sufficient information for survival prediction, we see the potential of combining multi-modality data for this critical task and have included a discussion on this interesting future research direction.

## 8 Conclusions

Pre-training techniques are hot research topics in ML and DL. It has attracted much attention in medical domain due to the challenges posed by medical data, such as the data scarcity and lack of annotation. We review in detail the recent advances in pre-training-based frameworks for healthcare areas. This work proposes suggestions for physicians and researchers in AI who want to learn about the latest pre-training techniques in the medical domain. We briefly introduced the publicly available medical benchmark datasets and general pre-training strategies. Sections 3–6 investigate the extensive use of pre-training in different scenarios in the medical domain from four perspectives: images, bio-signal data, EHR data, and multi-modality data. At the end of this survey, we discuss the challenges and their possible solutions.

## Acknowledgements

This work was supported by 2021 UQ School of Information Technology and Electrical Engineering (ITEE) Research Support Funding, Cyber Research Seed Funding (No. 2021-R3), the University of Adelaide (No. 1531570) and New Staff Research Start-up Funds (No. NS-2102).

## Open Access

This article is licensed under a Creative Commons Attribution 4.0 International License, which permits use, sharing, adaptation, distribution and reproduction in any medium or format, as long as you give appropriate credit to the original author(s) and the source, provide a link to the Creative Commons licence, and indicate if changes were made.

The images or other third party material in this article are included in the article′s Creative Commons licence, unless indicated otherwise in a credit line to the material. If material is not included in the article′s Creative Commons licence and your intended use is not permitted by statutory regulation or exceeds the permitted use, you will need to obtain permission directly from the copyright holder.

To view a copy of this licence, visit http://creativecommons.org/licenses/by/4.0/.

1109/LSP.2021.3114119.

[193] K. Radhika, V. R. M. Oruganti. Transfer learning for subject-independent stress detection using physiological signals. In *Proceedings of the 17th IEEE India Council International Conference*, New Delhi, India, 2020. DOI: 10.1109/INDICON49873.2020.9342505.

[194] P. Sarkar, S. Lobmaier, B. Fabre, D. González, A. Mueller, M. G. Frasch, M. C. Antonelli, A. Etemad. Detection of maternal and fetal stress from the electrocardiogram with self-supervised representation learning. *Scientific Reports*, vol. 11, no. 1, Article number 24146, 2021. DOI: 10.1038/S41598-021-03376-8.

[195] P. Sarkar, A. Etemad. Self-supervised ECG representation learning for emotion recognition. *IEEE Transactions on Affective Computing*, vol. 13, no. 3, pp. 1541–1554, 2022. DOI: 10.1109/TAFFC.2020.3014842.

[196] P. J. Aston, J. V. Lyle, E. Bonet-Luz, C. L. Huang, Y. M. Zhang, K. Jeevaratnam, M. Nandi. Deep learning applied to attractor images derived from ECG signals for detection of genetic mutation. In *Proceedings of Computing in Cardiology*, IEEE, Singapore, 2019. DOI: 10.22489/CinC.2019.097.

[197] Y. Cimtay, E. Ekmekcioglu. Investigating the use of pretrained convolutional neural network on cross-subject and cross-dataset EEG emotion recognition. *Sensors*, vol. 20, no. 7, Article number 2034, 2020. DOI: 10.3390/s20072034.

[198] S. Bagherzadeh, K. Maghooli, A. Shalbaf, A. Maghsoudi. Recognition of emotional states using frequency effective connectivity maps through transfer learning approach from electroencephalogram signals. *Biomedical Signal Processing and Control*, vol. 75, Article number 103544, 2022. DOI: 10.1016/j.bspc.2022.103544.

[199] M. N. Mohsenvand, M. R. Izadi, P. Maes. Contrastive representation learning for electroencephalogram classification. In *Proceedings of the Machine Learning for Health*, pp. 238–253, 2020.

[200] S. Raghu, N. Sriraam, Y. Temel, S. V. Rao, P. L. Kubben. EEG based multi-class seizure type classification using convolutional neural network and transfer learning. *PMLR Neural Networks*, vol. 124, pp. 202–212, 2020. DOI: 10.1016/j.neunet.2020.01.017.

[201] H. S. Nogay, H. Adeli. Detection of epileptic seizure using pretrained deep convolutional neural network and transfer learning. *European Neurology*, vol. 83, no. 6, pp. 602–614, 2020. DOI: 10.1159/000512985.

[202] S. Y. Tang, J. Dunnmon, K. K. Saab, X. Zhang, Q. Y. Huang, F. Dubost, D. Rubin, C. Lee-Messer. Self-supervised graph neural networks for improved electroencephalographic seizure analysis. In *Proceedings of the 10th International Conference on Learning Representations*, 2022.

[203] J. J. Xu, Y. J. Zheng, Y. F. Mao, R. X. Wang, W. S. Zheng. Anomaly detection on electroencephalography with self-supervised learning. In *Proceedings of IEEE International Conference on Bioinformatics and Biomedicine*, Seoul, Republic of Korea, pp. 363–368, 2020. DOI: 10.1109/BIBM49941.2020.9313163.

[204] H. Banville, O. Chehab, A. Hyvärinen, D. A. Engemann, A. Gramfort. Uncovering the structure of clinical EEG signals with self-supervised learning. *Journal of Neural Engineering*, vol. 18, no. 4, Article number 046020, 2021. DOI: 10.1088/1741-2552/abca18.

[205] M. T. Sadiq, M. Z. Aziz, A. Almogren, A. Yousaf, S. Siuly, A. U. Rehman. Exploiting pretrained CNN models for the development of an EEG-based robust BCI framework. *Computers in Biology and Medicine*, vol. 143, Article number 105242, 2022. DOI: 10.1016/j.compbiomed.2022.105242.

[206] Y. H. Ou, S. Q. Sun, H. T. Gan, R. Zhou, Z. Yang. An improved self-supervised learning for EEG classification. *Mathematical Biosciences and Engineering*, vol. 19, no. 7, pp. 6907–6922, 2022. DOI: 10.3934/mbe.2022325.

[207] H. Phan, O. Y. Chén, P. Koch, Z. Q. Lu, I. McLoughlin, A. Mertins, M. De Vos. Towards more accurate automatic sleep staging via deep transfer learning. *IEEE Transactions on Biomedical Engineering*, vol. 68, no. 6, pp. 1787–1798, 2021. DOI: 10.1109/TBME.2020.3020381.

[208] X. Jiang, J. H. Zhao, B. Du, Z. Y. Yuan. Self-supervised contrastive learning for EEG-based sleep staging. In *Proceedings of International Joint Conference on Neural Networks*, IEEE, Shenzhen, China, 2021. DOI: 10.1109/IJCNN52387.2021.9533305.

[209] N. Wagh, J. H. Wei, S. Rawal, B. M. Berry, L. Barnard, B. Brinkmann, G. Worrell, D. Jones, Y. Varatharajah. Domain-guided self-supervision of EEG data improves downstream classification performance and generalizability. In *Proceedings of Machine Learning for Health*, pp. 130–142, 2021.

[210] R. W. Picard, E. Vyzas, J. Healey. Toward machine emotional intelligence: Analysis of affective physiological state. *IEEE Transactions on Pattern Analysis and Machine Intelligence*, vol. 23, no. 10, pp. 1175–1191, 2001. DOI: 10.1109/34.954607.

[211] D. R. Simkin, R. W. Thatcher, J. Lubar. Quantitative EEG and neurofeedback in children and adolescents: Anxiety disorders, depressive disorders, comorbid addiction and attention-deficit/hyperactivity disorder, and brain injury. *Child and Adolescent Psychiatric Clinics of North America*, vol. 23, no. 3, pp. 427–464, 2014. DOI: 10.1016/j.chc.2014.03.001.

[212] G. Z. Zhao, Y. Ge, B. Y. Shen, X. J. Wei, H. Wang. Emotion analysis for personality inference from EEG signals. *IEEE Transactions on Affective Computing*, vol. 9, no. 3, pp. 362–371, 2017. DOI: 10.1109/TAFFC.2017.2786207.

[213] N. Lu, T. F. Li, X. D. Ren, H. Y. Miao. A deep learning scheme for motor imagery classification based on restricted boltzmann machines. *IEEE Transactions on Neural Systems and Rehabilitation Engineering*, vol. 25, no. 6, pp. 566–576, 2017. DOI: 10.1109/TNSRE.2016.2601240.

[214] M. McDermott, B. Nestor, E. Kim, W. C. Zhang, A. Goldenberg, P. Szolovits, M. Ghassemi. A comprehensive EHR timeseries pre-training benchmark. In *Proceedings of the Conference on Health, Inference, and Learning*, ACM, pp. 257–278, 2021. DOI: 10.1145/3450439.3451877.

[215] H. Chen, S. M. Lundberg, G. Erion, J. H. Kim, S. I. Lee. Forecasting adverse surgical events using self-supervised transfer learning for physiological signals. *Digital Medicine*, vol. 4, no. 1, Article number 167, 2021. DOI: 10.1038/s41746-021-00536-y.

[216] X. Xu, X. Xu, Y. Y. Sun, X. S. Liu, X. Li, G. T. Xie, F. Wang. Predictive modeling of clinical events with mutual enhancement between longitudinal patient records and medical knowledge graph. In *Proceedings of IEEE International Conference on Data Mining*, Auckland, New

Zealand, pp. 777–786, 2021. DOI: 10.1109/ICDM51629.2021.00089.

[217] Y. Xue, N. Du, A. Mottram, M. Seneviratne, A. M. Dai. Learning to select best forecast tasks for clinical outcome prediction. In *Proceedings of the 34th Conference on Neural Information Processing Systems*, Vancouver, Canada, pp. 15031–15041, 2020.

[218] S. Tipirneni, C. K. Reddy. Self-supervised transformer for sparse and irregularly sampled multivariate clinical time-series. *ACM Transactions on Knowledge Discovery from Data*, vol. 1, no. 1, Article number 105, 2022. DOI: 10.1145/3516367.

[219] H. X. Ren, J. Y. Wang, W. X. Zhao, N. Wu. RAPT: Pre-training of time-aware transformer for learning robust healthcare representation. In *Proceedings of the 27th ACM SIGKDD Conference on Knowledge Discovery & Data Mining*, Singapore, pp. 3503–3511, 2021. DOI: 10.1145/3447548.3467069.

[220] B. van Aken, J. M. Papaioannou, M. Mayrdorfer, K. Budde, F. Gers, A. Löser. Clinical outcome prediction from admission notes using self-supervised knowledge integration. In *Proceedings of the 16th Conference of the European Chapter of the Association for Computational Linguistics*, pp. 881–893, 2021. DOI: 10.18653/v1/2021.eacl-main.75.

[221] C. Lu, C. K. Reddy, Y. Ning. Self-supervised graph learning with hyperbolic embedding for temporal health event prediction. *IEEE Transactions on Cybernetics*, to be published. DOI: 10.1109/TCYB.2021.3109881.

[222] K. Hur, J. Lee, J. Oh, W. Price, Y. Kim, E. Choi. Unifying heterogeneous electronic health records systems via text-based code embedding. In *Proceedings of Conference on Health, Inference, and Learning*, pp. 183–203, 2022.

[223] S. Biswal, C. Xiao, L. M. Glass, E. Milkovits, J. M. Sun. Doctor2Vec: Dynamic doctor representation learning for clinical trial recruitment. *Proceedings of AAAI Conference on Artificial Intelligence*, vol. 34, no. 1, pp. 557–564, 2020. DOI: 10.1609/aaai.v34i01.5394.

[224] Y. P. Chen, Y. H. Lo, F. P. Lai, C. H. Huang. Disease concept-embedding based on the self-supervised method for medical information extraction from electronic health records and disease retrieval: Algorithm development and validation study. *Journal of Medical Internet Research*, vol. 23, no. 1, Article number e25113, 2021. DOI: 10.2196/25113.

[225] E. Lehman, S. Jain, K. Pichotta, Y. Goldberg, B. C. Wallace. Does BERT pretrained on clinical notes reveal sensitive data? In *Proceedings of Conference of the North American Chapter of the Association for Computational Linguistics: Human Language Technologies*, pp. 946–959, 2021. DOI: 10.18653/v1/2021.naacl-main.73.

[226] X. Y. Zhang, C. Xiao, L. M. Glass, J. M. Sun. DeepEnroll: Patient-trial matching with deep embedding and entailment prediction. In *Proceedings of The Web Conference*, ACM, Taipei, China, pp. 1029–1037, 2020. DOI: 10.1145/3366423.3380181.

[227] H. D. Hlynsson, S. Ellertsson, J. F. Daðason, E. L. Sigurdsson, H. Loftsson. Semi-self-supervised automated ICD coding. [Online], Available: https://arxiv.org/abs/2205.10088, 2022.

[228] Z. Zhang, J. S. Liu, N. Razavian. BERT-XML: Large scale automated ICD coding using BERT pretraining. In *Proceedings of the 3rd Clinical Natural Language Processing Workshop*, pp. 24–34, 2020. DOI: 10.18653/v1/2020.clinicalnlp-1.3.

[229] Y. Q. Su, Y. L. Shi, W. Lee, L. Cheng, H. M. Guo. TAHDNet: Time-aware hierarchical dependency network for medication recommendation. *Journal of Biomedical Informatics*, vol. 129, Article number 104069, 2022. DOI: 10.1016/j.jbi.2022.104069.

[230] J. Y. Shang, T. F. Ma, C. Xiao, J. M. Sun. Pre-training of graph augmented transformers for medication recommendation. In *Proceedings of the 28th International Joint Conference on Artificial Intelligence*, Macao, China, pp. 5953–5959, 2019. DOI: 10.24963/ijcai.2019/825.

[231] Z. Sun, S. L. Peng, Y. N. Yang, X. Q. Wang, F. Li. A general fine-tuned transfer learning model for predicting clinical task acrossing diverse EHRs datasets. In *Proceedings of IEEE International Conference on Bioinformatics and Biomedicine*, San Diego, USA, pp. 490–495, 2019. DOI: 10.1109/BIBM47256.2019.8983098.

[232] L. T. Ma, X. Y. Ma, J. Y. Gao, X. F. Jiao, Z. H. Yu, C. H. Zhang, W. J. Ruan, Y. S. Wang, W. Tang, J. T. Wang. Distilling knowledge from publicly available online EMR data to emerging epidemic for prognosis. In *Proceedings of Web Conference*, ACM, Ljubljana, Slovenia, pp. 3558–3568, 2021. DOI: 10.1145/3442381.3449855.

[233] H. Quan, V. Sundararajan, P. Halfon, A. Fong, B. Burnand, J. C. Luthi, L. D. Saunders, C. A. Beck, T. E. Feasby, W. A. Ghali. Coding algorithms for defining comorbidities in ICD-9-CM and ICD-10 administrative data. *Medical Care*, vol. 43, no. 11, pp. 1130–1139, 2005. DOI: 10.1097/01.mlr.0000182534.19832.83.

[234] Y. Y. Zhang, X. Wu, Q. Fang, S. S. Qian, C. S. Xu. Knowledge-enhanced attributed multi-task learning for medicine recommendation. *ACM Transactions on Information Systems*, to be published. DOI: 10.1145/3527662.

[235] Y. K. Li, H. Y. Wang, Y. Luo. A comparison of pre-trained vision-and-language models for multimodal representation learning across medical images and reports. In *Proceedings of IEEE International Conference on Bioinformatics and Biomedicine*, Seoul, Republic of Korea, pp. 1999–2004, 2020. DOI: 10.1109/BIBM49941.2020.9313289.

[236] L. H. Li, M. Yatskar, D. Yin, C. J. Hsieh, K. W. Chang. VisualBERT: A simple and performant baseline for vision and language. [Online], Available: https://arxiv.org/abs/1908.03557, 2019.

[237] Y. C. Chen, L. J. Li, L. C. Yu, A. El Kholy, F. Ahmed, Z. Gan, Y. Cheng, J. J. Liu. UNITER: UNiversal Image-TExt representation learning. [Online], Available: https://arxiv.org/abs/1909.11740, 2019.

[238] H. Tan, M. Bansal. LXMERT: Learning cross-modality encoder representations from transformers. In *Proceedings of Conference on Empirical Methods in Natural Language Processing and the 9th International Joint Conference on Natural Language Processing*, Hong Kong, China, pp. 5100–5111, 2019. DOI: 10.18653/v1/D19-1514.

[239] Z. C. Huang, Z. Y. Zeng, B. Liu, D. M. Fu, J. L. Fu. Pixel-BERT: Aligning image pixels with text by deep multi-modal transformers. [Online], Available: https://arxiv.org/abs/2004.00849, 2020.

[240] Y. Khare, V. Bagal, M. Mathew, A. Devi, U. D. Priyakumar, C. V. Jawahar. MMBERT: Multimodal BERT pretraining for improved medical VQA. In *Proceedings of the 18th IEEE International Symposium on Biomedical Imaging*, Nice, France, pp. 1033–1036, 2021. DOI: 10.1109/ISBI48211.2021.9434063.

[241] N. Rieke, J. Hancox, W. Q. Li, F. Milletarì, H. R. Roth, S. Albarqouni, S. Bakas, M. N. Galtier, B. A. Landman, K. Maier-Hein, S. Ourselin, M. Sheller, R. M. Summers, A. Trask, D. G. Xu, M. Baust, M. J. Cardoso. The future of digital health with federated learning. *Digital Medicine*, vol. 3, no. 1, Article number 119, 2020. DOI: 10.1038/s41746-020-00323-1.

[242] M. Abadi, A. Chu, I. Goodfellow, H. B. McMahan, I. Mironov, K. Talwar, L. Zhang. Deep learning with differential privacy. In *Proceedings of ACM SIGSAC Conference on Computer and Communications Security*, Vienna, Austria, pp. 308–318, 2016. DOI: 10.1145/2976749.2978318.

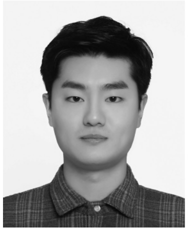

**Yixuan Qiu** received the M. Sc. degree in electrical engineering from The University of Queensland, Australia in 2020. Currently, he is a Ph. D. degree candidate in data science at School of information Technology and Electrical Engineering, The University of Queensland, Australia.

His research interests include medical data analytic, self-supervised learning and federated learning.

E-mail: y.qiu@uq.edu.au

RCID iD: 0000-0002-7593-1876

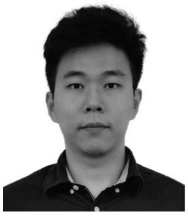

**Feng Lin** received the M. Sc. degree from The University of Queensland, Australia in 2022. He is currently working at Wipro, Australia.

His research interests include weakly-supervised learning, data mining and deep learning.

E-mail: feng.lin@uq.net.au

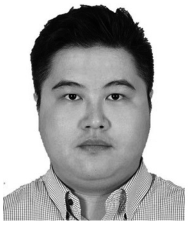

**Weitong Chen** received the Ph. D. degree in computer science from The University of Queensland, Australia in 2020. He is currently a lecturer at The University of Adelaide, Australia.

His research interests include machine learning and its application to medical domains.

E-mail: t.chen@adelaide.edu.au (Corresponding author)

ORCID iD: 0000-0003-1001-7925

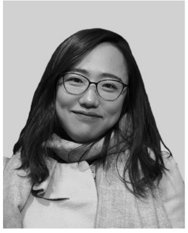

**Miao Xu** received the Ph. D. degree in machine learning from Nanjing University, China in 2017. She is a lecturer at The University of Queensland (UQ), Australia. Before joining UQ, she was a postdoctoral researcher at RIKEN, Japan.

Her research interests include weakly supervised learning and its application to the medical and cyber-security domains.

E-mail: miao.xu@uq.edu.au
